# Micro-computed Tomography-Based Collagen Orientation and Anisotropy Analysis of Rabbit Articular Cartilage

**DOI:** 10.1007/s10439-023-03183-4

**Published:** 2023-04-01

**Authors:** Simo P. Ojanen, Mikko A. J. Finnilä, Walter Herzog, Simo Saarakkala, Rami K. Korhonen, Lassi Rieppo

**Affiliations:** 1 Department of Technical Physics, University of Eastern Finland, P.O. Box 1627, 70210 Kuopio, Finland; 2 Research Unit of Health Sciences and Technology, University of Oulu, Oulu, Finland; 3 Human Performance Laboratory, Faculty of Kinesiology, University of Calgary, Calgary, AB Canada; 4 Department of Diagnostic Radiology, Oulu University Hospital, Oulu, Finland

**Keywords:** Anterior cruciate ligament transection, Animal model, Early osteoarthritis, Hexamethyldisilazane, Structure tensor analysis, Polarized light microscopy

## Abstract

The collagen network is the highly organized backbone of articular cartilage providing tissue tensile stiffness and restricting proteoglycan bleaching out of the tissue. Osteoarthritis (OA) diminishes proper collagen network adaptation. Our aim was to provide quantitative three-dimensional (3D) information of the cartilage collagen network adaptation in early osteoarthritis using high resolution micro-computed tomography (*µ*CT)-imaging. Osteochondral samples from the femoral condyles were collected from healthy (*N* = 8, both legs) and experimental OA rabbit model with anterior cruciate ligament transection (*N* = 14, single leg). Samples were processed for cartilage *µ*CT-imaging and histological evaluation with polarized light microscopy (PLM). Structure tensor analysis was used to analyse the collagen fibre orientation and anisotropy of the *µ*CT-images, and PLM was used as a validation for structural changes. Depth-wise comparison of collagen fibre orientation acquired with *µ*CT-imaging and PLM correlated well, but the values obtained with PLM were systematically greater than those measured with *µ*CT-imaging. Structure tensor analysis allowed for 3D quantification of collagen network anisotropy. Finally, *µ*CT-imaging revealed only minor differences between the control and experimental groups.

**Supplementary Information:**

The online version contains supplementary material available at 10.1007/s10439-023-03183-4.

## Introduction

Articular cartilage (AC) is a soft tissue found at the ends of long bones in diarthrodial joints, providing near frictionless contact and distributing the applied load, thus minimizing stress to subchondral bone.^3^ AC is composed of water (~ 75%), different macromolecules and chondrocytes. AC extracellular matrix (ECM) consists the framework of structural macromolecules.^3,36^ Chondrocytes, *i.e.*, cartilage cells, are responsible for the maintenance of the ECM. The primary macromolecules of the cartilage ECM are proteoglycans and collagen, mostly type II collagen.^8,22^ Type II collagen is a fibril forming protein, which forms a network within the ECM. Mature cartilage is structurally divided into four depth-wise regions: superficial, transitional (middle), radial (deep), and calcified cartilage zone. The collagen fibrils in mature cartilage are oriented in an arc like manner, that is, they run parallel to the surface in the superficial zone and gradually turn to run perpendicular to the surface in the deep zone. Collagen fibres are anchored to the underlaying subchondral bone through the calcified cartilage. The ECM is highly anisotropic in the superficial and deep zones but has been considered essentially isotropic in the middle zone.

Osteoarthritis (OA) is the most common disease of the knee and is associated with pain, joint stiffness, inflammation and swelling, and in the mid- and end-stages affects the quality of life. OA is associated with a degradation of the collagen network, depletion of proteoglycans, and an increase in water content in AC. These structural changes, in turn, reduce the biomechanical properties of the cartilage and result in functional impairments of the joint.^4,23^ Anterior cruciate ligament transection (ACLT) is commonly used pre-clinical model of post-traumatic OA for the study of the onset and progression OA. ACLT in rabbits has been shown to cause changes in cartilage structure and composition, and alter the biomechanical properties of cartilage and chondrocytes as early as two weeks post-ACLT.^26^

The collagen network orientation and organization is typically evaluated from thin histological sections imaged with polarized light microscopy (PLM).^1,30^ Collagen network anisotropy has been determined using magnetic resonance imaging,^9,24,40^ PLM,^18,30^ scanning electron microscopy,^39^ second harmonic generation microscopy,^21^ and Fourier-transform infrared spectroscopy.^42^ The microscopic methods are often limited to two-dimensional (2D) analysis, and magnetic resonance imaging has an inherently poor resolution making it unfeasible for detailed microstructural analysis. Micro-computed tomography (*µ*CT) has excellent spatial resolution and is a relatively fast imaging method, which makes it useful for non-invasive evaluation of tissue structures. However, articular cartilage has poor contrast in *µ*CT, thus, contrast agents that bind to proteoglycans/glycosaminoglycans^2,10,14,19,35^ or collagen^25^ are required to achieve detailed structural information. Drying of soft tissue samples in the presence of hexamethyldisilazane (HMDS) has been used to enhance the natural contrast in *µ*CT-imaging without contrast agents^16,17,32^ to provide three-dimensional (3D) structural information with high resolution. Structure tensor analysis and eigenvalue decomposition allows for the quantitative evaluation of orientation and anisotropy from 2D images and 3D image stacks.^37^ Structure tensor is derived from the gradient of the grey scales of an image or a 3D image stack, where the gradient distribution is described with respect to the neighbourhood of the observation point. In 3D, the gradient distribution can be decomposed to eigenvalues (λ_1_, λ_2_, λ_3_) and the corresponding eigenvectors (*e*_1_, *e*_2_, *e*_3_), in which λ_1_ > λ_2_ > λ_3_. The eigenvalue decomposition describes the local gradient characteristics and can be visualized as 3D ellipses. These features can determine the orientation and anisotropy of the observed sub-volumes of the image stack and the entire volume of interest can be mapped for local orientation and anisotropy information.^37^ The smallest eigenvalue (λ_3_) reflects the minimum variability of the structure, which is taken as the principal direction of anisotropy, and it corresponds to the local fibre orientation for the analysed voxel.^37^ The degree of anisotropy is calculated as value one minus the ratio of the smallest to the highest eigenvalue (1−*λ*_3_/*λ*_1_) and is considered to be 0 for perfectly isotropic material and 1 for anisotropic material.^37^ Structure tensor analysis is widely used in image processing and has been used in the medical research for instance to determine the fibre anisotropy and orientation from rat brain histological sections,^5^ the anisotropy of trabecular bone in 3D,^38^ and the collagen orientation of HMDS-prepared human meniscus in 3D.^15^

In this study, we used structure tensor analysis in combination with *µ*CT-imaging of HMDS-prepared rabbit AC samples. We compared the results of the structure tensor analysis-based collagen fibre orientation values to PLM, which is considered the gold standard for AC collagen orientation mapping. Validation was done with healthy control animals. Secondarily, we investigated if early post-traumatic OA caused by ACLT induces changes in the depth-wise orientation and anisotropy of the collagen fibril network. Based on previous studies,^15,32,37^ we hypothesized that the structure tensor analysis can be used to quantify the orientation and anisotropy of the collagen fibres. Additionally, we expected little difference in the orientation and anisotropy of the collagen network in the superficial zone of this early post-traumatic OA rabbit model compared to healthy control.

## Materials and Methods

**Figure 1:**
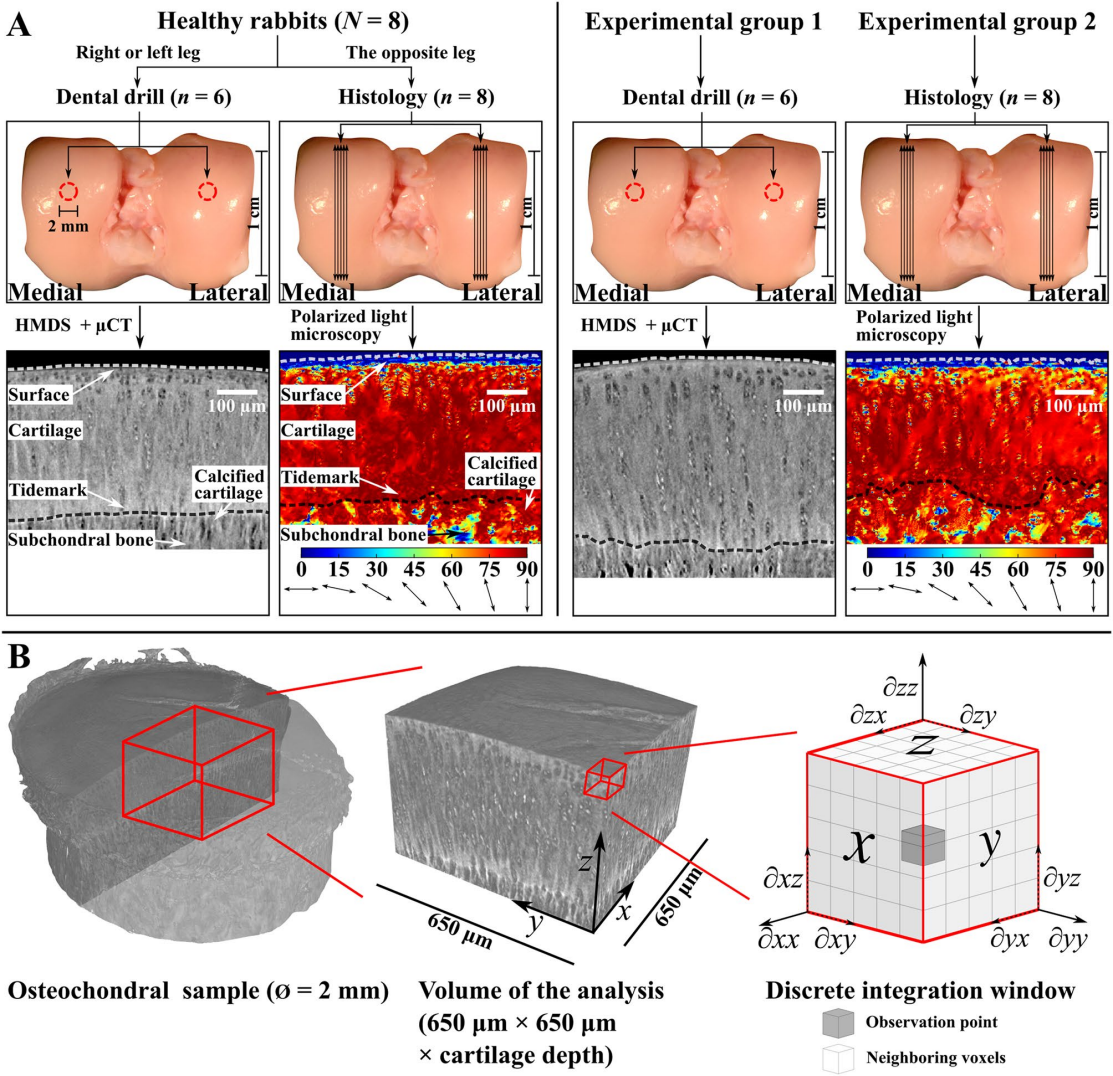
(a) Samples from eight healthy rabbits were prepared such, that a randomly picked leg was prepared for histology (polarized light microscopy, *n* = 8) and the other one for *µ*CT imaging (*n* = 6). Two experimental groups went through anterior cruciate ligament transection: the other group was prepared for histology (polarized light microscopy, *n* = 8) and the other one for *µ*CT imaging (*n* = 6). (b) Illustration of the HMDS processed *µ*CT imaged osteochondral sample (left). 650 *µ*m × 650 *µ*m × cartilage thickness volume of the analysis was chosen approximately from the centre of the osteochondral plug (diameter 2 mm) (centre). A discrete cubic integration window (right) was used for the structure tensor analysis of the volume of interest to investigate the structural gradients of the volume of interest in the *x*-, *y*- and *z*-directions. The observation point is at the centre of the integration window to with respect to which the gradient from the surrounding voxels is calculated. The gradient kernel is determined by radius size of the integration window. The entire volume of interest is mapped stepwise with the integration window to evaluate the local gradients.

Osteochondral samples were collected from the lateral and medial femoral condyles of both knees from eight (*N* = 8, 16 knees) healthy skeletally mature New Zealand white rabbits (*Oryctolagus cuniculus*, age 12.5 months at the time of sample harvesting, weight 4.57 ± 0.35 kg) (Fig. 1a). In addition, a unilateral ACLT was performed on fourteen (*N* = 14) rabbits (age 12 months at the time of surgery, weight 4.44 ± 0.45 kg). The experimental rabbits were sacrificed two weeks post-surgery, and the lateral and medial femoral condyles from each rabbit’s experimental leg were harvested. The operated knee was chosen randomly to avoid side-to-side bias.

The experimental rabbits were divided into two groups for further analysis. The first group (*n* = 6) was prepared for *µ*CT-imaging and the second group (*n* = 8) for histological analysis. Samples from the healthy control group were prepared such that a random knee joint from each rabbit was prepared for *µ*CT-imaging (*n* = 6) and the other one for histology (*n* = 8). Knee joints from the control group rabbits were picked randomly (left/right) to randomize possible side-to-side effects.

All procedures were carried out according to the guidelines of the Canadian Council on Animal Care and were approved by the committee on Animal Ethnics at the University of Calgary.

Osteochondral plugs (diameter 2 mm) from the healthy (lateral, *n* = 6; medial, *n* = 6) and the experimental (lateral, *n* = 6; medial, *n* = 6) knee joints were extracted from the central load bearing areas of the lateral and medial femoral condyles with a dental trephine drill (TRE020M, Ortomat Herpola Ltd, Turku, Finland) (Fig. 1a). Six knee joints from the control group rabbits were used for *µ*CT-imaging. Osteochondral samples were then frozen in phosphate buffered saline (PBS). After thawing, samples were fixed by immersing in cacodylate buffered 1.3% (v/v) glutaraldehyde containing 0.7% (w/v) Hexaammineruthenium(III) chloride (RHT) and decalcified with ethylenediaminetetraacetic (EDTA). Samples were then dehydrated using a series of ethanol of increasing concentrations (30, 50, 70, 80, 90, 96, 100%). Finally, samples were immersed into a Hexamethyldisilazane (HMDS, Sigma) for 2 h and air-dried at room temperature.

Detailed sample preparation for the histology can be found in Ojanen *et al*.^26^ Briefly, samples were fixed in formalin, decalcified with EDTA, dehydrated, and embedded in paraffin. Histological sections from the most central bearing area of the lateral and medial femoral condyles from the healthy (lateral, *n* = 8; medial, *n* = 7) and experimental (lateral, *n* = 8; medial, *n* = 8) group rabbits were prepared for PLM perpendicular to the AC surface (sagittal plane, section thickness 5 *µ*m, 2 slides/sample, totalling 64 slides, enzymatic removal of proteoglycans).

The HMDS-processed samples were imaged with a desktop *µ*CT-scanner (SkyScan 1272, Bruker microCT, Kontich, Belgium) using the following settings: tube voltage 40 kV; tube current 250 *µ*A; no additional filtration; isotropic voxel size 0.65 *µ*m; number of projections 1800; averaging 5 frames/projection; exposure time 1300 ms; scan time ~ 4 h. The reconstructions were made with NRecon software (version 1.7.0.4, Bruker microCT). Furthermore, Gaussian smoothing, ring-artifact correction and beam-hardening correction were applied.

**Figure 2:**
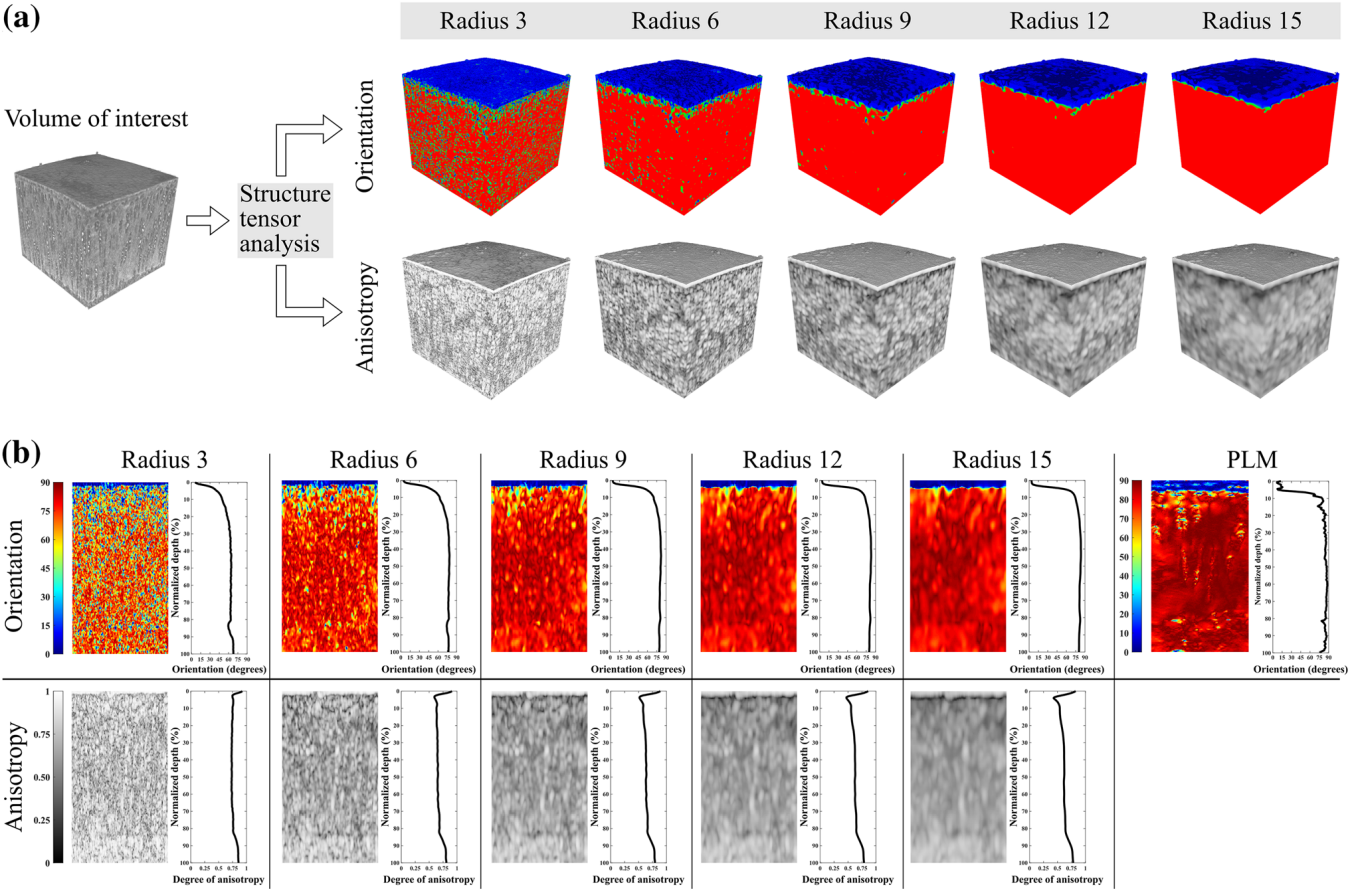
(a) Visualization of the effect of the used kernel radius size to the orientation and the anisotropy analyses. The used radius sizes in the study were 3, 6, 9, 12, 15 voxels. (b) Example views in a *x–z*-plane of the normalized orientation and the anisotropy analyses, and the corresponding depth-wise average profiles from a healthy rabbit cartilage sample collected from the medial femoral condyle (VOI: 500 *µ*m × 500 *µ*m × cartilage thickness). Average profile for each sample was calculated from the normalized image stack (normalization to 200 points from the surface to the bottom of the cartilage). Each image slice (*x*–*y*-plane) was averaged to represent the value for a certain depth (*z*-direction). Illustration of a normalized polarized light microscopy (PLM) image (200 points from the surface to the bottom of the cartilage) collected from the medial femoral condyle of a healthy rabbit, and the corresponding depth-wise profile is presented on the right-hand side.

Structure tensor analysis can be used to determine the local orientation and anisotropy of material matrix.^37^ In this analysis, the principal directions of the structure tensor are calculated (as gradients in x-, y- and z-directions) from the original grey value images. A discrete integration window determines the volume unit in which the local orientation and anisotropy are calculated. The image stack is mapped with this integration window to define the orientation and anisotropy distribution throughout the sample (Fig. 1b). The actual size of the integration window is determined as 2 × radius + 1 pixel.^37^ In this study, cubic integration windows with radius sizes of 3, 6, 9, 12 and 15 were used (Fig. 2a), resulting in window size diameters of 7, 13, 19, 25 and 31 voxels, respectively. Several integration window sizes were used to determine the optimal window size. The range of the window sizes was influenced by a previous study focusing on the same method applied to evaluate collagen orientation in human menisci, which used an integration window of 19 voxels (isotropic imaging voxel size of 2.0 *µ*m).^15^

**Figure 3:**
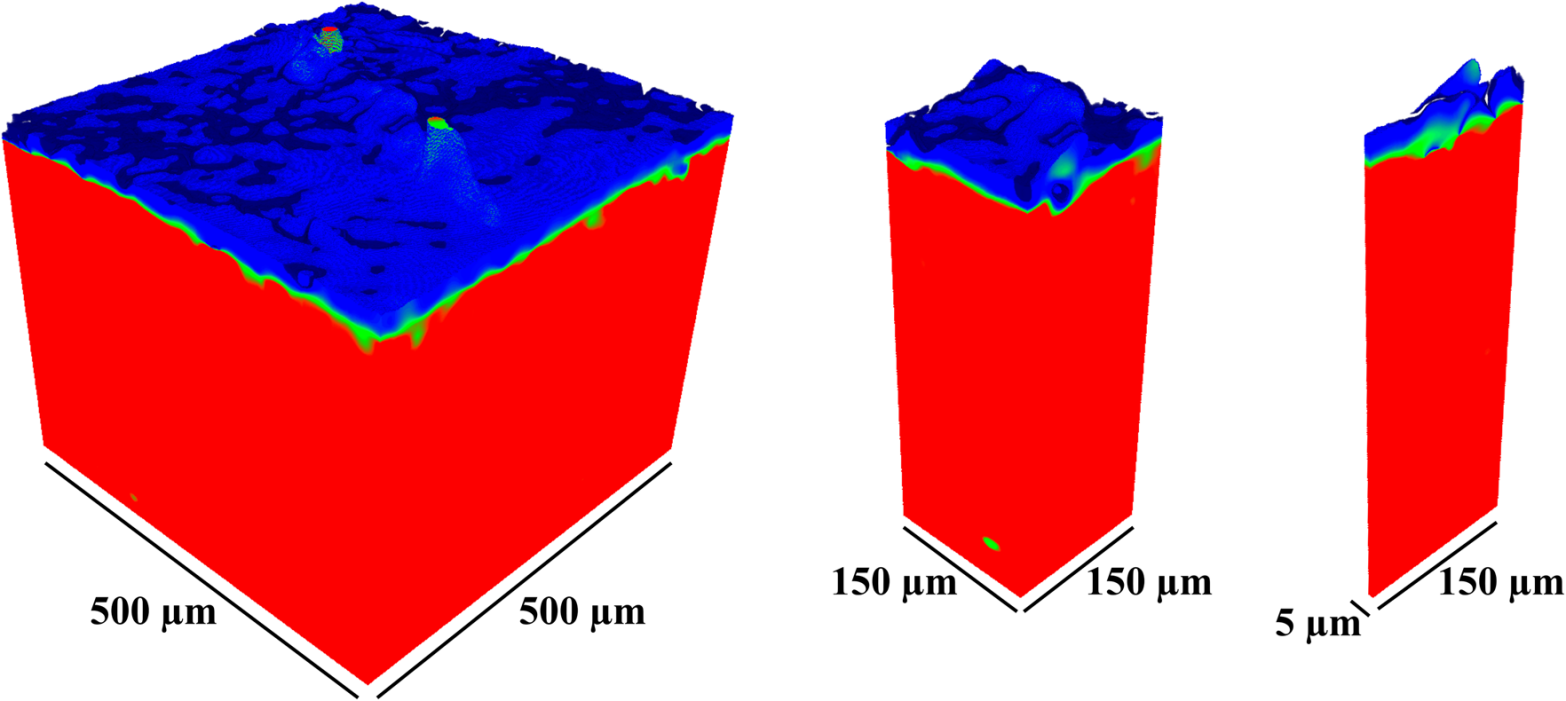
Visualization of the three volumes of interest used to the orientation analyses (integration window radius 12) of the healthy medial femoral cartilage. From left to right VOI: 500 *µ*m × 500 *µ*m × cartilage thickness, 150 *µ*m × 150 *µ*m × cartilage thickness, and 150 *µ*m × 5 *µ*m × cartilage thickness. The identical VOIs were used for the anisotropy analyses.

From the reconstructed image stacks, a 650 *µ*m × 650 *µ*m × cartilage depth (1000 × 1000 × cartilage depth as voxels) sized volume of interest (VOI) was chosen for the structure tensor analysis. First, a 3D gradient was calculated for the volume of the integration window and filtered with 3D Gaussian filter. Subsequently, an eigenvalue decomposition was applied to the structure tensor, which produces three eigenvalues and the corresponding eigenvectors.^37^ The resulting anisotropy and orientation stacks were masked with a binary mask (all voids were filled, and the surface was smoothed with dilation) acquired from the original *µ*CT-image stack with Otsu’s thresholding method.^28^ Each sample was thresholded separately resulting in individual image thresholds for each image stack. A new VOI of 500 *µ*m × 500 *µ*m × cartilage depth (770 × 770 × cartilage depth as voxels), the same as used in a pilot study,^32^ was determined to eliminate the boundary artifacts caused by the integration window being partially outside the image stack at the edges. Furthermore, VOI was cropped at the bottom (from the direction of subchondral bone) before further analysis to eliminate the above-mentioned artifact. The interface between air and cartilage prevents the artificial boundary at the cartilage surface. Additionally, smaller VOI of 150 *µ*m × 150 *µ*m and 150 *µ*m × 5 *µ*m were also analysed to reflect the region of interest of the PLM imaging (details below) (Fig. 3a). All the VOIs were interpolated into 200 points from the detected surface to the bottom of the VOI and an average value was calculated for each interpolation step to acquire a depth-wise profile (Fig. 2b). All analyses were performed with custom-developed MATLAB scripts (R2020b, The MathWorks, Inc., Natick, MA, USA).

PLM was used to acquire the collagen network organization of the cartilage ECM.^30,31^ Light microscopy (Nikon Diaphot TMD, Nikon Inc. Shinagawa, Tokyo, Japan, pixel size = 1.03 *µ*m) with an Abrio PLM system (Cri, Inc., Woburn, MA, USA) was used for depth-wise collagen orientation mapping of the histological samples.^26^ In the PLM figures, the pixel value (0–90) represents the orientation, such that 0° is parallel and 90° is perpendicular to the cartilage surface (Fig. 1a). For the depth-wise orientation analysis, a region of interest (150 *µ*m × cartilage depth) was chosen, the image was interpolated into 200 points in a depth-wise manner, and laterally averaged to acquire one value for each normalized depth (Fig. 2b).

Dataset normality (of the averaged profile groups) for each depth-wise step was tested using the Shapiro–Wilk test with a level of significance of *p* = 0.05. The anisotropy and orientation profiles of the healthy and the experimental groups for each integration window size were compared in a point-by-point manner using Mann–Whitney *U*-testing, since normal distribution for each analysed depth could not be assured. The agreement of the depth-wise orientation profiles obtained using PLM and the structure tensor analysis of the healthy rabbit AC was assessed using a Pearson’s correlation analysis and Bland–Altman plots. The normality of the data used in the Bland–Altman analysis (observations of PLM reduced from CT) was not confirmed with Shapiro–Wilk testing, and consequently the percentiles of 2.5 and 97.5 were chosen to represent the spread of the data. All statistical analyses were performed with custom-written MATLAB scripts (R2020b, The MathWorks, Inc., Natick, MA, USA).

## Results

**Figure 4:**
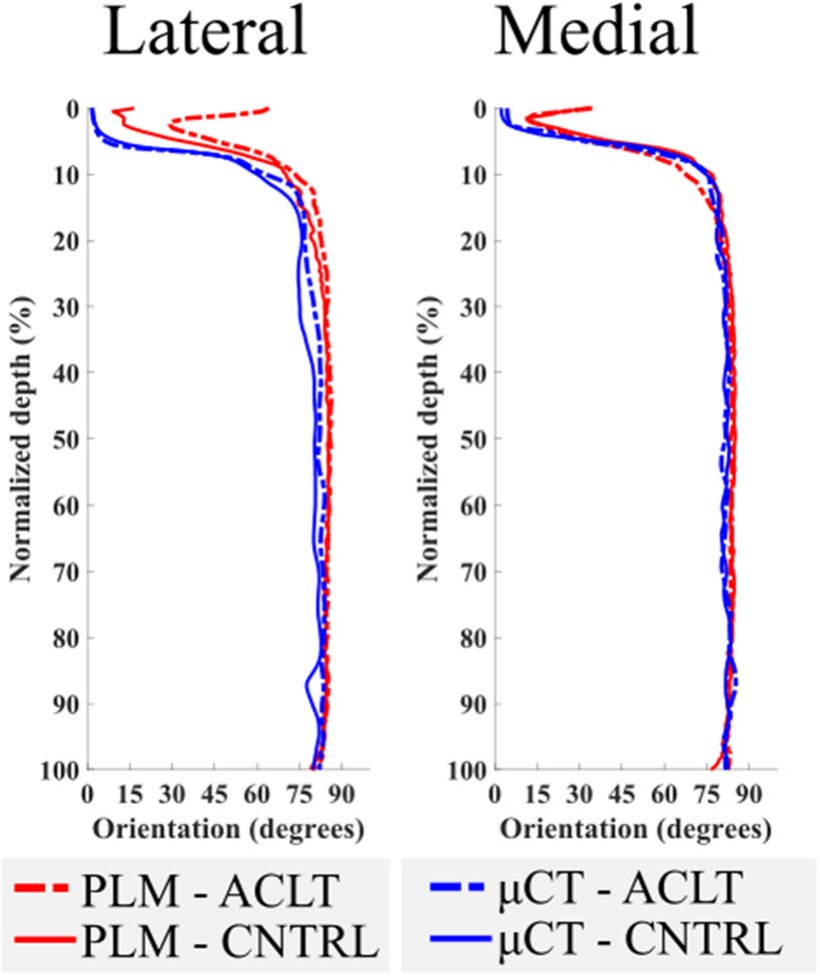
Average depth-wise orientation profiles of the structure tensor analysis (integration window radius 15, VOI: 150 *µ*m × 5 *µ*m × cartilage thickness, blue lines) and the polarized light microscopy (PLM, red lines) of the lateral and medial femoral condyle cartilage of the healthy (CNTRL) and the experimental (ACLT) rabbit knee joints.

The integration window size used in the structure tensor analysis influenced the orientation and anisotropy parameters in a depth-wise manner (Figures S1–S6). Orientation angles gradually increased especially at the normalized depth of 10–100% in all analysed groups when the integration window size was increased (Figures S1–S3). Moreover, the orientation angles obtained using great integration window sizes (radius 9, 12 and 15) did not differ much from each other, but the small radius window sizes (radius 3 and 6) produced differences in the collagen fibril orientation angles (Figures S1–S3). The large integration windows gave small anisotropy throughout most of the cartilage (5–100% cartilage depth) (Figures S4–S6). Visual inspection of the orientation and anisotropy image stacks revealed greater detail when using small integration window sizes and increasing the integration window size resulted in a more homogenous structure (Fig. 2). Moreover, visual inspection of the collagen orientation acquired with the structure tensor analysis (integration window radius size 15) and PLM revealed an arc-shaped collagen structure and general agreement, but they were not absolutely the same, especially in the superficial cartilage zone (Figs. 4 and S7).

**Figure 5:**
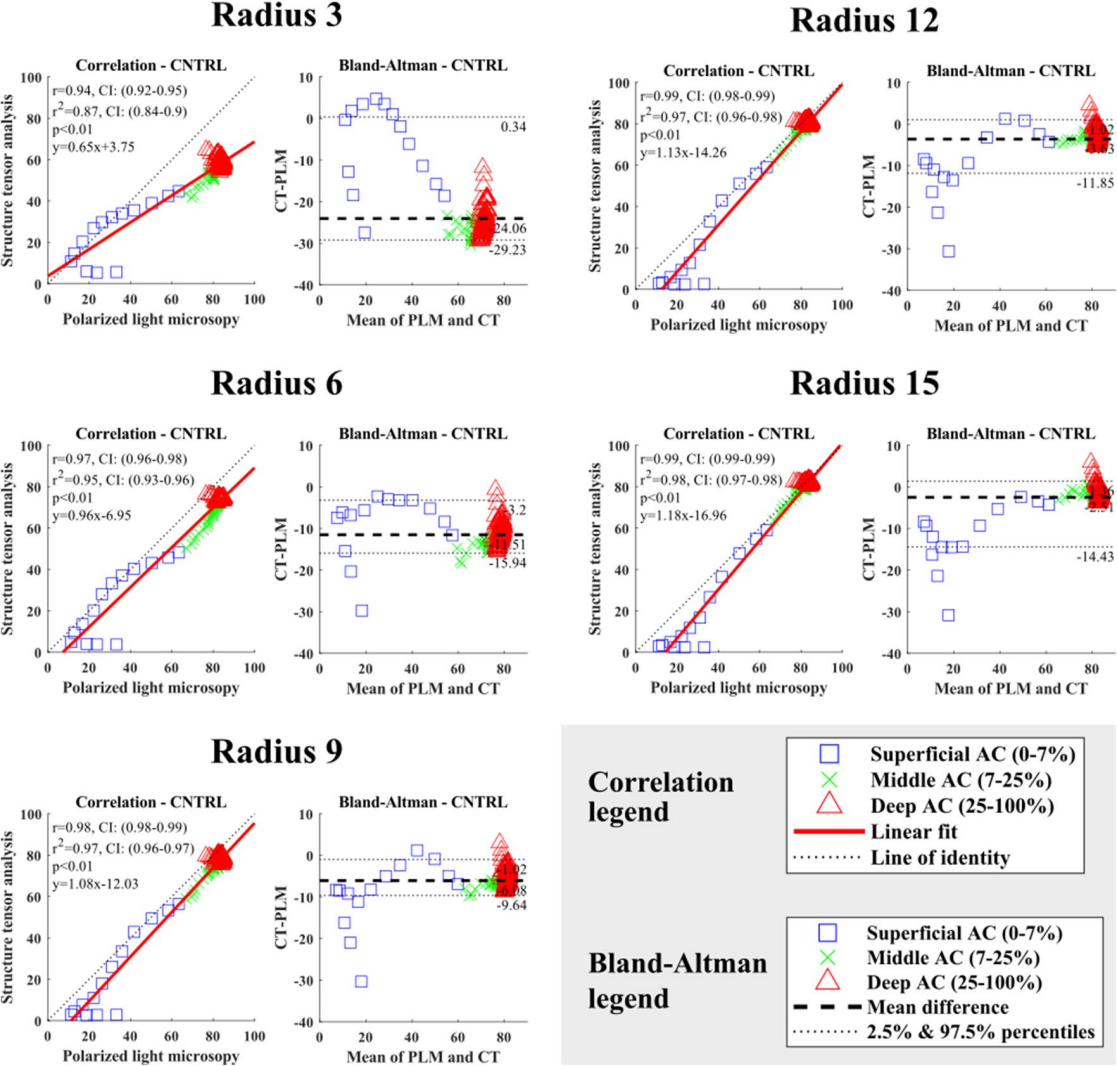
The correlation and the Bland–Altman analyses of the cartilage extracellular matrix orientation acquired with polarized light microscopy (PLM) and structure tensor analysis (VOI: 150 *µ*m × 5 *µ*m × cartilage thickness) of the medial femoral condyle cartilage from the healthy rabbit knee joints. The analyses are made to the normalized data. The PLM analysis is compared to the different radius sized used in the structure tensor analysis. Articular cartilage is divided into three sections in a depth-wise manner for illustrative purposes: superficial cartilage (0–7%); middle of cartilage (7–25%); deep cartilage (25–100%). This division was not used in the correlation nor Bland–Altman analyses.

**Figure 6:**
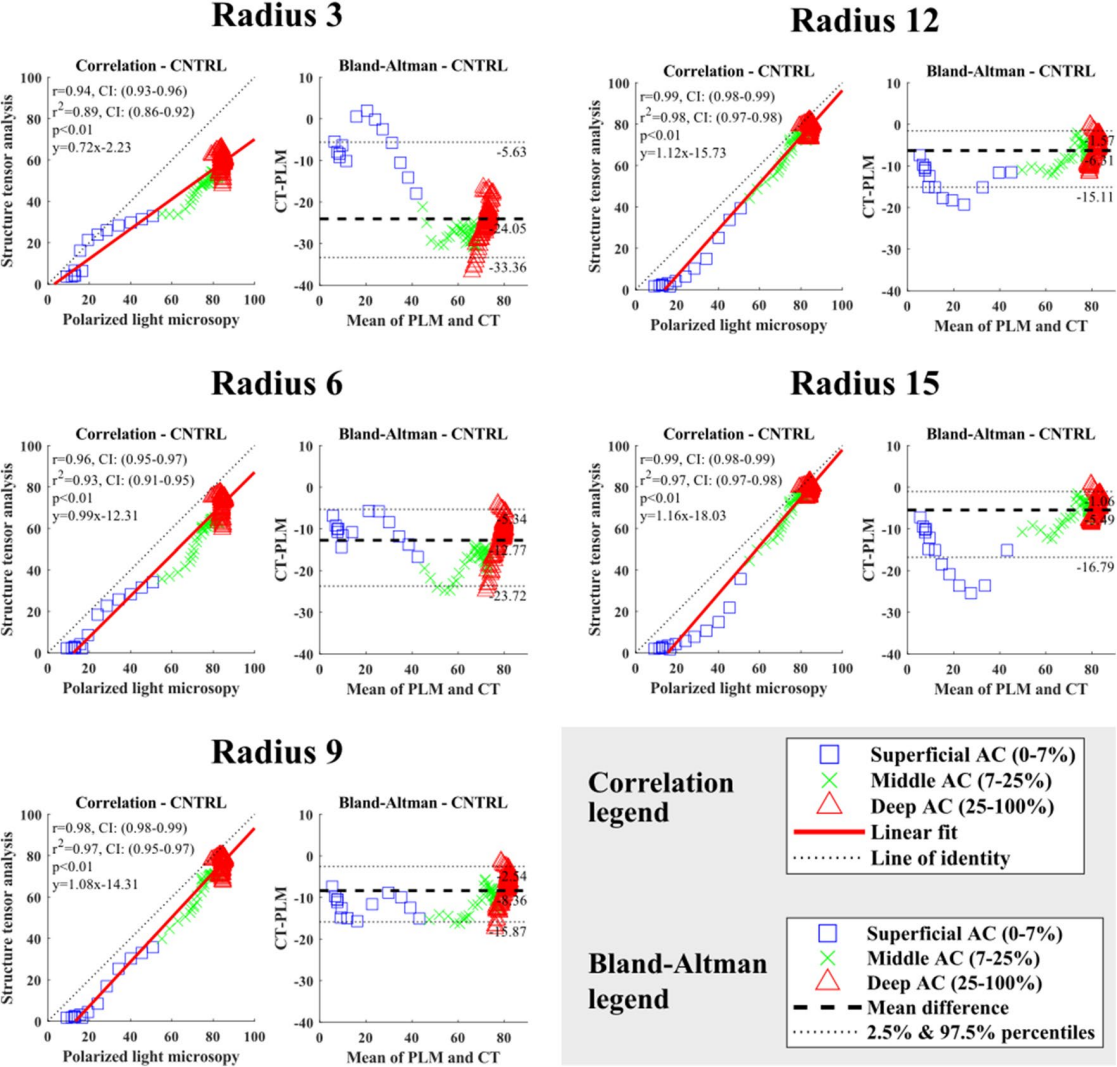
The correlation and the Bland–Altman analyses of the cartilage extracellular matrix orientation acquired with polarized light microscopy (PLM) and structure tensor analysis (VOI: 150 *µ*m × 5 *µ*m × cartilage thickness) of the lateral femoral condyle cartilage from the healthy rabbit knee joints. The analyses are made to the normalized data. The PLM analysis is compared to the different radius sized used in the structure tensor analysis. Articular cartilage is divided into three sections in a depth-wise manner for illustrative purposes: superficial cartilage (0–7%); middle of cartilage (7–25%); deep cartilage (25–100%). This division was not used in the correlation nor Bland–Altman analyses.

As expected, the depth-wise collagen orientation angles increased with depth for all integration window sizes (Figures S1–S3). Correlation between PLM and structure tensor analysis-based orientation angles was excellent (*r* ≥ 0.95) with all integration window sizes used in both the lateral and medial femoral condyles in the healthy knee joints, except for the VOI of 150 *µ*m × 5 *µ*m × cartilage thickness (Figs. 5, 6 and S8–S11). The Bland–Altman analysis supported this result (Figs. 5, 6 and S8–S11, Tables 1, S1 and S2). The difference of the mean/median between the PLM and structure tensor analysis decreased with increasing integration window size (Tables 1, S1 and S2). However, the mean and median differences (CT-PLM) were systematically negative. Increasing the integration window from 3 to 12 decreased the variance (CT-PLM) between the cumulative percentiles 2.5 and 97.5%, meaning less spread of the distribution (Tables 1, S1 and S2). Nevertheless, with an integration window radius of 15, the spread of the difference (CT-PLM) was greater than with an integration window radius of 9 and 12. Visual inspection of the Bland–Altman plots revealed that the greatest variance and most outliers are located in the superficial most layers of the samples (~ 0–3% of cartilage depth), while for the remaining depth (~ 3–100% of cartilage depth) the collagen fibres are packed tightly around the mean and within the analysed percentiles (Figs. 5, 6 and S8–S11). Increasing the radius size decreased the spread of the Bland–Altman plots.

**Table 1:** The Bland–Altman analyses of the cartilage extracellular matrix orientation acquired with polarized light microscopy and structure tensor analysis (VOI: 150 *µ*m × 5 *µ*m) of the lateral and medial femoral condyle cartilage from the healthy rabbit knee joints.

Lateral Femoral Condyle						
Difference (CT-PLM)						
Radius	Mean	Median	STD	2.50%	97.50%	Range
3	− 24.05	− 24.92	6.01	− 33.36	− 5.63	27.73
6	− 12.77	− 11.56	4.42	− 23.72	− 5.34	18.37
9	− 8.36	− 7.76	3.49	− 15.87	− 2.54	13.34
12	− 6.31	− 5.47	3.38	− 15.11	− 1.57	13.54
15	− 5.49	− 4.67	3.94	− 16.79	− 1.06	15.74

Medial femoral condyle						
Difference (CT-PLM)						
Radius	Mean	Median	STD	2.50%	97.50%	Range
3	− 24.06	− 25.61	5.99	− 29.23	0.34	29.58
6	− 11.51	− 11.77	3.18	− 15.94	− 3.20	12.74
9	− 6.08	− 6.29	2.98	− 9.64	− 1.02	8.62
12	− 3.63	− 3.56	3.25	− 11.85	1.02	12.87
15	− 2.51	− 2.21	3.57	− 14.43	1.36	15.80

*CT* computed tomography, *PLM* polarized light microscopy, *STD* standard deviation, *2.50%* Value of the cumulative percentile at 2.50%,*97.50%* Value of the cumulative percentile at 97.50%, *Range* 2.5% reduced from 97.5%

Anisotropy of the collage fibres reached a maximum in the superficial cartilage (~ 0–2% of cartilage depth) and was followed by a rapid decrease (~ 4–7% of cartilage depth) (Figs. 3 and S4–S6). The bigger the integration window size, the greater the difference between these two superficial depth regions. Beyond depths of about 7%, collagen fibril anisotropy gradually increased and then remained almost constant for the rest of the cartilage depth (Figures S4-S6). Moreover, another rapid decrease in anisotropy of collagen fibres was found in the deep cartilage (~ 90–94% of cartilage depth), which may be caused by the cartilage tidemark. Beyond the tidemark, anisotropy increased again. When comparing these “landmarks” of the anisotropy profiles to the orientation profiles of traditional cartilage zones, the maximum anisotropy occurred in the presumed superficial zone and the first decrease in anisotropy in the presumed transitional zone (~ 30°–50° of orientation) of cartilage, indicating greater anisotropy in the superficial and less anisotropy in the middle zone cartilage. Moreover, the second decrease in anisotropy (at the assumed tidemark) is consistent in both the depth-wise anisotropy and the orientation analyses.

The collagen orientation angles obtained from the structure tensor analysis of the healthy and the experimental knee joints showed small differences in the deep zone of the cartilage (~ 90–94% of cartilage depth) on the medial femoral condyles when using the radius sizes of 3 and 6 in the VOI of 500 *µ*m × 500 *µ*m × cartilage thickness (Figure S3). Group-wise differences of the collagen orientation occurred in the deep cartilage (~ 90–94% of cartilage depth) with almost all radius sizes in the lateral and medial femoral condyles, and differences were also found using radius sizes of 12 (~ 30–45% of cartilage depth) and 15 (~ 30–55% of cartilage depth) in the lateral femoral condyle in the VOI of 150 *µ*m × 150 *µ*m × cartilage thickness (Figure S2). Some differences in collagen orientation between the control and experimental cartilage samples were identified with almost all radius sizes in the lateral and medial femoral condyles at varying depths in the VOI of 150 *µ*m × 5 *µ*m × cartilage thickness (Figure S1). The depth-wise anisotropy analysis between the experimental and healthy cartilage differed slightly around the assumed tidemark only for some of the VOIs and for selected radius sizes (Figures S4–S6).

## Discussion

Our study shows that structure tensor analysis applied to *µ*CT-imaging data of HMDS-processed tissue specimen can be used to quantitatively evaluate the rabbit articular cartilage collagen orientation and anisotropy in 3D, which both reflect the collagen network configuration and condition. This conclusion is supported by the strong correlation with the reference orientation angle acquired with the PLM, which is considered the gold standard for cartilage and other soft tissue structural orientation.^1,30,33,41^ Further, we demonstrated that the integration window size influences the results of the structure tensor analysis, and thus, in this case, the resulting collagen fibre network orientation and anisotropy. The greatest correlation between the structure tensor analysis-based orientation and the orientation acquired with PLM was achieved with kernel sizes 12 and 15.

We found slight changes in collagen orientation between healthy control and ACL-transected experimental group samples using PLM (Figure S11).^26^ The *µ*CT-based structure tensor analysis revealed these same differences when using the VOI of 150 *µ*m × 150 *µ*m × cartilage thickness and 150 *µ*m × 5 *µ*m × cartilage thickness in the lateral femoral condyle cartilage (Figures S1 and S2) but did not reveal these differences when using the VOI of 500 *µ*m × 500 *µ*m × cartilage thickness (Figure S3). Visual inspection of the depth-wise profiles from the different VOIs used in this study varied between, with the small VOIs having more fluctuation in the profiles highlighting local changes in the collagen fibril network of the cartilage ECM. This finding highlights the difference between a more local compared to a more global analysis, and the benefits of an adjustable volume analysis of *µ*CT-imaging. A *µ*CT-image stack can readily be divided into several local volumes or can be analysed as a global entity (Figure S12). In future studies, it would be beneficial to analyse samples from all locations of the knee and analyse changes in the structural ECM components longitudinally with a number of time points, as we have done using other methods than used here.^11,13,26,27^

Apart from the similarities and the strong correlation between the orientation analyses of the structure tensor and PLM, the differences between the obtained results are more than expected since the methods are based on totally different physical principles. While PLM is based on the interaction of the tissue with polarized light, the structure tensor analysis relies on grayscale differences between neighbouring image voxels. Also, PLM is based on two-dimensional histological analysis, which may be associated with preparation artifacts in the analysis area.^34^ Once a histological sample is processed, it cannot be changed and provides information only in that plane. In the *µ*CT-analysis, there is great flexibility and the VOIs can be chosen to suit the purpose and imaging and reconstruction artifacts can be minimized. Most importantly, our *µ*CT method preserves the 3D structure of the sample, providing a more global analysis of the tissue than section-based PLM.

Structure tensor analysis showed the superficial cartilage to be highly anisotropic followed by a less anisotropic region, and again a more anisotropic region in the deep cartilage, which agrees with previous studies.^24,30,40,42^ This result is consistent with the known collagen fibre orientation in cartilage which is highly organized in the superficial and deep zone cartilage where most of the fibres are oriented in the same direction. In the middle zone cartilage, collagen fibrils are more randomly oriented resulting in an essentially homogenous structure,^30,40^ which agrees with our results. Moreover, below the tidemark, anisotropy increased suggesting a more oriented calcified cartilage. Since cartilage is anisotropic in the depth and the transversal plane, it is important to evaluate cartilage structure three-dimensionally, which cannot be done easily using histology. Previously, anisotropy of cartilage had been successfully studied with magnetic resonance imaging,^9,24,40^ PLM^18,30^ and Fourier-transform infrared imaging.^42^ Magnetic resonance imaging is limited by its poor spatial and temporal resolution, and histological and PLM evaluation is typically performed to 2D histological sections. Here, we showed that structure tensor analysis can be used to determine the 3D orientation and anisotropy of the collagen network with high resolution. In future studies, it would be important to compare the results obtained using structure tensor analysis of cartilage anisotropy to all the other methods mentioned above.

Structure tensor analysis is widely used in image processing^37^ and has been applied to study histological sections of rat brains,^5^ trabecular bone,^38^ human menisci,^15^ and cartilage.^32^ However, structure tensor analysis-based anisotropy was not used in studies on articular cartilage.^32^ Benefits of the structure tensor analysis are relatively easy implementation since it is based on the gradient of images, and its applicability to 3D data. However, structure tensor analysis of large volumes is time consuming and requires great computational power. Integration window size significantly affected the *µ*CT-based collagen orientation and anisotropy values. Small integration windows detect local inhomogeneities of the cartilage structure, including chondrons, structural irregularities, and clefts, while large integration windows provide a more general view of the collagen fibril network. With small integration windows, our analysis took a few hours, while large windows required about 24 h for each sample. Global analysis of cartilage samples, a preferred option for further studies, would require even more time and more advanced computer performance than used here.

We found the greatest correlation between the *µ*CT and PLM with kernel sizes 12 and 15. The radius of the structure tensor needs to be adjusted according to the aims of the study. One should consider the spatial and contrast resolution as well as the size of the features and details that one wants to analyse. For small details and target features, a small kernel size is required, and for greater features, greater kernel sizes are likely optimal. In early OA, minor changes in cartilage structure and function are difficult to identify. In order to detect small changes in collagen orientation, a small integration window size is likely to be beneficial. However, we found only minor differences in the collagen orientation between early OA and healthy cartilage samples with almost all radius sizes. We can only speculate whether alterations would have appeared in advanced OA samples and with which radius size they would have been found. This subject needs further investigation.

There are many limitations regarding to this study. Even though *µ*CT-imaging and HMDS sample processing has great potential for structural analyses of soft tissues,^15–17,32^ results can suffer from traditional *µ*CT-related artefacts, such as ring and beam hardening. In this study, ring artefacts could largely be avoided by selecting appropriate VOI but might still have caused small variations in the results.

Structure tensor analysis was conducted to image stacks with background noise. For the final analysis, orientation and anisotropy stacks were masked by surface recognition based on Otsu thresholding^28^ of the original *µ*CT-image stacks. The surface of the original *µ*CT-image stacks and the final analysis stacks might thus not be identical, possibly leading to variations of results in surface and superficial cartilage. The locations of the VOI (*µ*CT) and regions of interest (PLM) were chosen visually. Specifically, the bottom end of the VOI was chosen at the subchondral bone-calcified cartilage-interface, thus including some part of the subchondral bone. For the PLM analysis, the region of interest was chosen from the cross-sectional retardation and orientation images, where features of subchondral bone started to appear. This may have caused differences in results for the bottom most ~ 90–100% of the sample thickness, where calcified cartilage and subchondral bone might have affected the lateral averaging.

Differences between the structure tensor analysis and PLM might also be caused by inter animal variations as the healthy knee joints for histology and *µ*CT-imaging were taken from the same rabbit while the experimental joints came from two different animals. Optimally, the samples for *µ*CT-imaging and histology would be prepared from the same joint. Bland–Altman analysis showed some differences between the methods, which might be highlighted by the fact that the samples were prepared from separate legs and not from the same sample.

Even though the overall anatomy of the rabbit knee is similar to that of the human knee,^20^ there are some notable differences in cartilage structure, composition, and function. Rabbit knee joint AC is thinner compared to human and the cell volume fraction, collagen network configuration, and proteoglycan content are different.^6,20,29^ Moreover, the depth-wise transition of collagen fibres is different between rabbit and human cartilage, since the transition from superficial to deep cartilage in rabbit cartilage may be more rapid compared to the transition in human cartilage.^7,12^ The results of this study are not transferable to human AC, but they underline the importance of structure tensor analysis and its applicability for studies of AC from different species.

Our study shows the potential of structure tensor analysis applied to the *µ*CT-imaged HMDS-processed AC samples, and its capability to quantify local orientation and anisotropy. Our analysis was able to identify subtle differences in the depth-wise AC organization in a very early post-traumatic OA model of rabbit.

## Supplementary Information

Below is the link to the electronic supplementary material.

Depth-wise orientation analysis of the lateral and medial femoral condyle cartilage of the healthy and the experimental groups acquired with structure tensor analysis of the µCT images (VOI: 150 µm × 5 µm × cartilage thickness) using different radius sizes (3, 6, 9, 12, 15). The data was normalized into 200 points in a depth-wise manner and each image slice (x-y-plane) was averaged laterally for a group-wise comparison. Red and blue lines represent the experimental and the healthy groups, respectively, and the shaded areas the corresponding 95% confidence intervals. The red dashed line represents the statistical difference (Mann-Whitney U-test) at the corresponding normalized depth of cartilage. Supplementary file1 (TIF 1455 kb)

Depth-wise orientation analysis of the lateral and medial femoral condyle cartilage of the healthy and the experimental groups acquired with structure tensor analysis of the µCT images (VOI: 150 µm × 150 µm × cartilage thickness) using different radius sizes (3, 6, 9, 12, 15). The data was normalized into 200 points in a depth-wise manner and each image slice (x-y-plane) was averaged laterally for a group-wise comparison. Red and blue lines represent the experimental and the healthy groups, respectively, and the shaded areas the corresponding 95% confidence intervals. The red dashed line represents the statistical difference (Mann-Whitney U-test) at the corresponding normalized depth of cartilage. Supplementary file2 (TIFF 997 kb)

Depth-wise orientation analysis of the lateral and medial femoral condyle cartilage of the healthy and the experimental groups acquired with structure tensor analysis of the µCT images (VOI: 500 µm × 500 µm × cartilage thickness) using different radius sizes (3, 6, 9, 12, 15). The data was normalized into 200 points in a depth-wise manner and each image slice (x-y-plane) was averaged laterally for a group-wise comparison. Red and blue lines represent the experimental and the healthy groups, respectively, and the shaded areas the corresponding 95% confidence intervals. The red dashed line represents the statistical difference (Mann-Whitney U-test) at the corresponding normalized depth of cartilage. Supplementary file3 (TIFF 955 kb)

Depth-wise anisotropy analysis of the lateral and medial femoral condyle cartilage of the healthy and the experimental groups acquired with structure tensor analysis of the µCT images (VOI: 150 µm × 5 µm × cartilage thickness) using different radius sizes (3, 6, 9, 12, 15). The data was normalized into 200 points in a depth-wise manner and each image slice (x-y-plane) was averaged laterally for a group-wise comparison. Red and blue lines represent the experimental and the healthy groups, respectively, and the shaded areas the corresponding 95% confidence intervals. The red dashed line represents the statistical difference (Mann-Whitney U-test) at the corresponding normalized depth of cartilage. Supplementary file4 (TIF 1031 kb)

Depth-wise anisotropy analysis of the lateral and medial femoral condyle cartilage of the healthy and the experimental groups acquired with structure tensor analysis of the µCT images (VOI: 150 µm × 150 µm × cartilage thickness) using different radius sizes (3, 6, 9, 12, 15). The data was normalized into 200 points in a depth-wise manner and each image slice (x-y-plane) was averaged laterally for a group-wise comparison. Red and blue lines represent the experimental and the healthy groups, respectively, and the shaded areas the corresponding 95% confidence intervals. The red dashed line represents the statistical difference (Mann-Whitney U-test) at the corresponding normalized depth of cartilage. Supplementary file5 (TIF 994 kb)

Depth-wise anisotropy analysis of the lateral and medial femoral condyle cartilage of the healthy and the experimental groups acquired with structure tensor analysis of the µCT images (VOI: 500 µm × 500 µm × cartilage thickness) using different radius sizes (3, 6, 9, 12, 15). The data was normalized into 200 points in a depth-wise manner and each image slice (x-y-plane) was averaged laterally for a group-wise comparison. Red and blue lines represent the experimental and the healthy groups, respectively, and the shaded areas the corresponding 95% confidence intervals. The red dashed line represents the statistical difference (Mann-Whitney U-test) at the corresponding normalized depth of cartilage. Supplementary file6 (TIF 991 kb)

Average depth-wise orientation profiles of the structure tensor analysis (integration window radius 15, VOIs: 150 µm × 150 µm × cartilage thickness, and 500 µm × 500 µm × cartilage thickness, blue lines) and the polarized light microscopy (PLM, red lines) of the lateral and medial femoral condyle cartilage of the healthy (CNTRL) and the experimental (ACLT) rabbit knee joints. B: Depth-wise orientation analysis of the lateral and medial femoral condyle cartilage of the healthy and the experimental groups acquired with polarized light microscopy. The data was normalized into 200 points in a depth-wise manner and averaged laterally for a group-wise comparison. Red and blue lines represent the experimental and the healthy groups, respectively, and the shaded areas the corresponding 95% confidence intervals. The red dashed line represents the statistical difference (Mann-Whitney U-test) at the corresponding normalized depth of cartilage. Supplementary file7 (TIF 2678 kb)

The correlation and the Bland-Altman analyses of the cartilage extracellular matrix orientation acquired with polarized light microscopy (PLM) and structure tensor analysis (VOI: 150 µm × 150 µm × cartilage thickness) of the medial femoral condyle cartilage from the healthy rabbit knee joints. The analyses are made to the normalized data. The PLM analysis is compared to the different radius sizes used in the structure tensor analysis. Articular cartilage is divided into three sections in a depth-wise manner for illustrative purposes: superficial cartilage (0-7%); middle of cartilage (7-25%); deep cartilage (25-100%). This division was not used in the correlation nor Bland-Altman analyses. Supplementary file8 (TIF 2912 kb)

The correlation and the Bland-Altman analyses of the cartilage extracellular matrix orientation acquired with polarized light microscopy (PLM) and structure tensor analysis (VOI: 150 µm × 150 µm × cartilage thickness) of the lateral femoral condyle cartilage from the healthy rabbit knee joints. The analyses are made to the normalized data. The PLM analysis is compared to the different radius sizes used in the structure tensor analysis. Articular cartilage is divided into three sections in a depth-wise manner for illustrative purposes: superficial cartilage (0-7%); middle of cartilage (7-25%); deep cartilage (25-100%). This division was not used in the correlation nor Bland-Altman analyses. Supplementary file9 (TIF 2912 kb)

The correlation and the Bland-Altman analyses of the cartilage extracellular matrix orientation acquired with polarized light microscopy (PLM) and structure tensor analysis (VOI: 500 µm × 500 µm × cartilage thickness) of the medial femoral condyle cartilage from the healthy rabbit knee joints. The analyses are made to the normalized data. The PLM analysis is compared to the different radius sizes used in the structure tensor analysis. Articular cartilage is divided into three sections in a depth-wise manner for illustrative purposes: superficial cartilage (0-7%); middle of cartilage (7-25%); deep cartilage (25-100%). This division was not used in the correlation nor Bland-Altman analyses. Supplementary file10 (TIFF 2912 kb)

The correlation and the Bland-Altman analyses of the cartilage extracellular matrix orientation acquired with polarized light microscopy (PLM) and structure tensor analysis (VOI: 500 µm × 500 µm × cartilage thickness) of the lateral femoral condyle cartilage from the healthy rabbit knee joints. The analyses are made to the normalized data. The PLM analysis is compared to the different radius sizes used in the structure tensor analysis. Articular cartilage is divided into three sections in a depth-wise manner for illustrative purposes: superficial cartilage (0-7%); middle of cartilage (7-25%); deep cartilage (25-100%). This division was not used in the correlation nor Bland-Altman analyses. Supplementary file11 (TIFF 2912 kb)

Example images and depth-wise orientation and anisotropy profiles of the structure tensor analysis (integration window radius 12) of a healthy medial femoral condyle cartilage. Left: volume of 500 µm × 500 µm × cartilage thickness is laterally divided into smaller volumes of 100 µm × 100 µm × cartilage thickness and the corresponding normalized depth-wise profiles of the orientation (up) and anisotropy (down) are presented from each small volume. Right: volume of 500 µm × 500 µm × cartilage thickness is divided into smaller volumes of 500 µm × 5 µm × cartilage thickness and the corresponding normalized depth-wise profiles of the orientation (up) and anisotropy (down) are presented from each small volume. These volume divisions are not used in this study, but they are examples of some of the numerous options of the analysis regions for further investigation. Supplementary file12 (TIFF 2835 kb)

The Bland-Altman analyses of the cartilage extracellular matrix orientation acquired with polarized light microscopy and structure tensor analysis (VOI: 150 µm × 150 µm × cartilage thickness) of the lateral and medial femoral condyle cartilage from the healthy rabbit knee joints. Supplementary file13 (DOCX 16 kb)

The Bland-Altman analyses of the cartilage extracellular matrix orientation acquired with polarized light microscopy and structure tensor analysis (VOI: 500 µm × 500 µm × cartilage thickness) of the lateral and medial femoral condyle cartilage from the healthy rabbit knee joints. Supplementary file14 (DOCX 16 kb)
