# Supplementary figures and images for: Micro-computed Tomography-Based Collagen Orientation and Anisotropy Analysis of Rabbit Articular Cartilage
Source: Ann Biomed Eng. 2023 Apr 1;51(8):1769–80. doi: 10.1007/s10439-023-03183-4 (PMC10326148; doi:10.1007/s10439-023-03183-4)

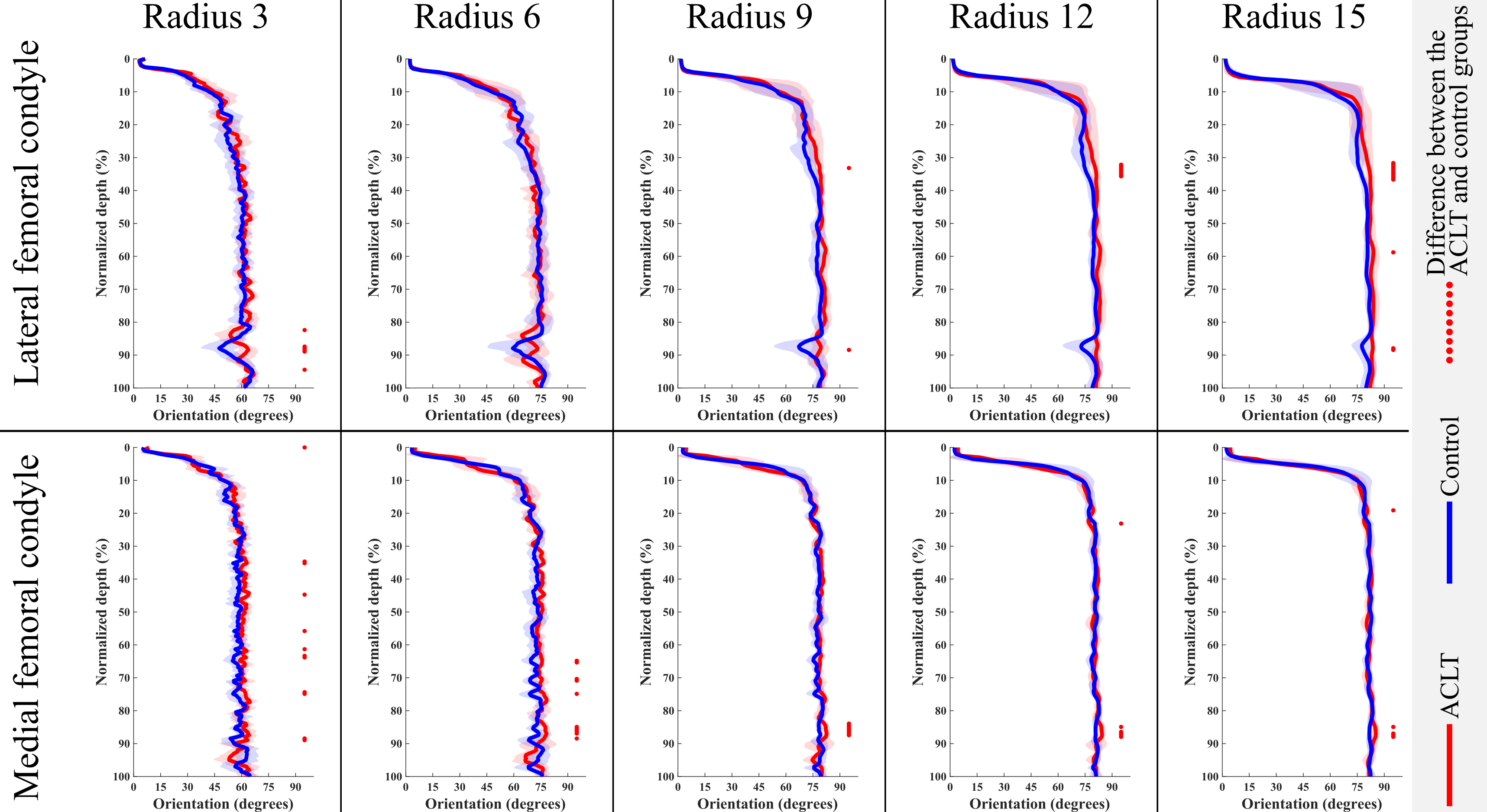

Supplement: Supplementary file 1 — Depth-wise orientation analysis of the lateral and medial femoral condyle cartilage of the healthy and the experimental groups acquired with structure tensor analysis of the µCT images (VOI: 150 µm × 5 µm × cartilage thickness) using different radius sizes (3, 6, 9, 12, 15). The data was normalized into 200 points in a depth-wise manner and each image slice (x-y-plane) was averaged laterally for a group-wise comparison. Red and blue lines represent the experimental and the healthy groups, respectively, and the shaded areas the corresponding 95% confidence intervals. The red dashed line represents the statistical difference (Mann-Whitney U-test) at the corresponding normalized depth of cartilage. Supplementary file1 (TIF 1455 kb) [file 10439_2023_3183_MOESM1_ESM.tif]

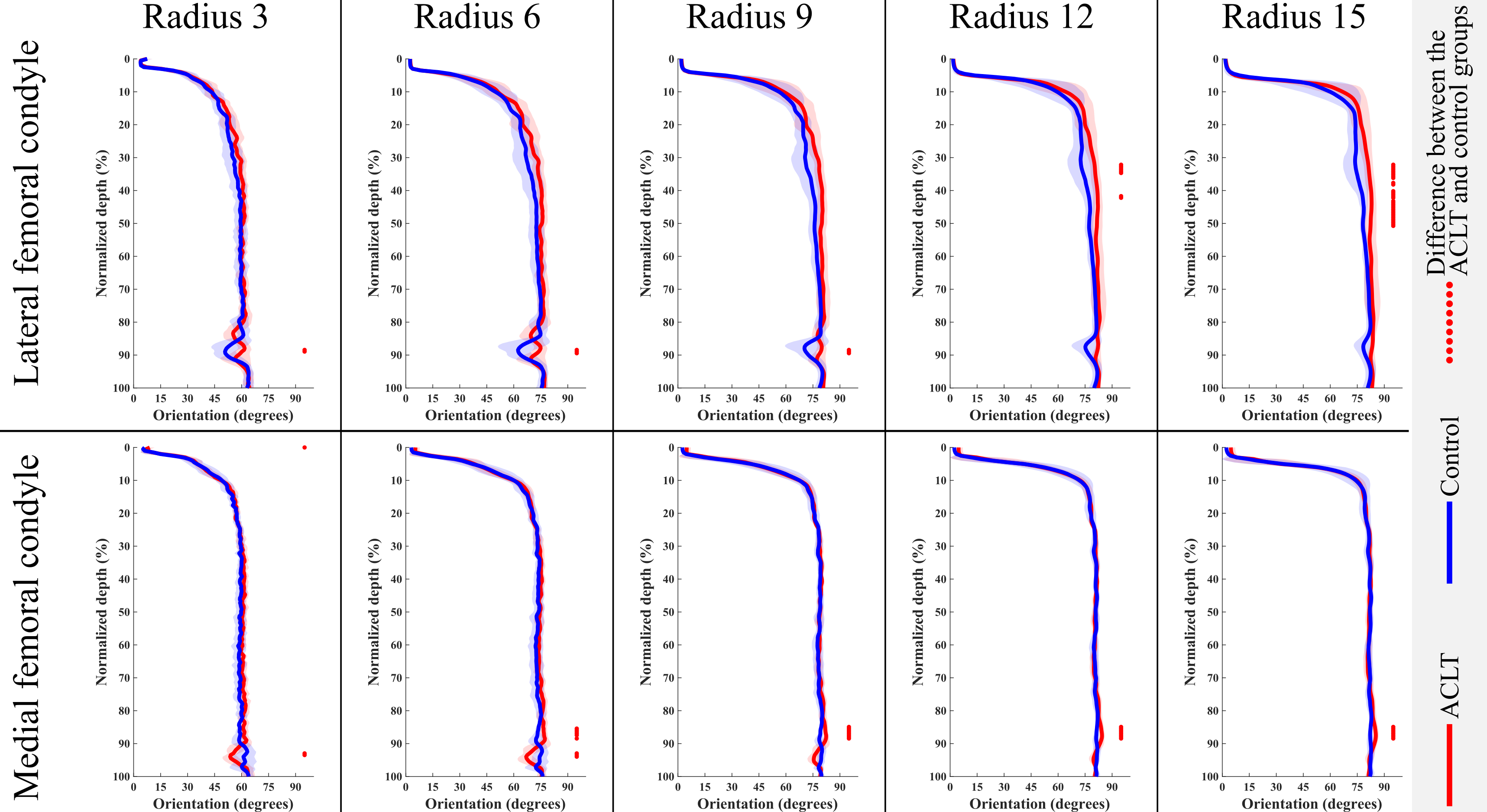

Supplement: Supplementary file 2 — Depth-wise orientation analysis of the lateral and medial femoral condyle cartilage of the healthy and the experimental groups acquired with structure tensor analysis of the µCT images (VOI: 150 µm × 150 µm × cartilage thickness) using different radius sizes (3, 6, 9, 12, 15). The data was normalized into 200 points in a depth-wise manner and each image slice (x-y-plane) was averaged laterally for a group-wise comparison. Red and blue lines represent the experimental and the healthy groups, respectively, and the shaded areas the corresponding 95% confidence intervals. The red dashed line represents the statistical difference (Mann-Whitney U-test) at the corresponding normalized depth of cartilage. Supplementary file2 (TIFF 997 kb) [file 10439_2023_3183_MOESM2_ESM.tif]

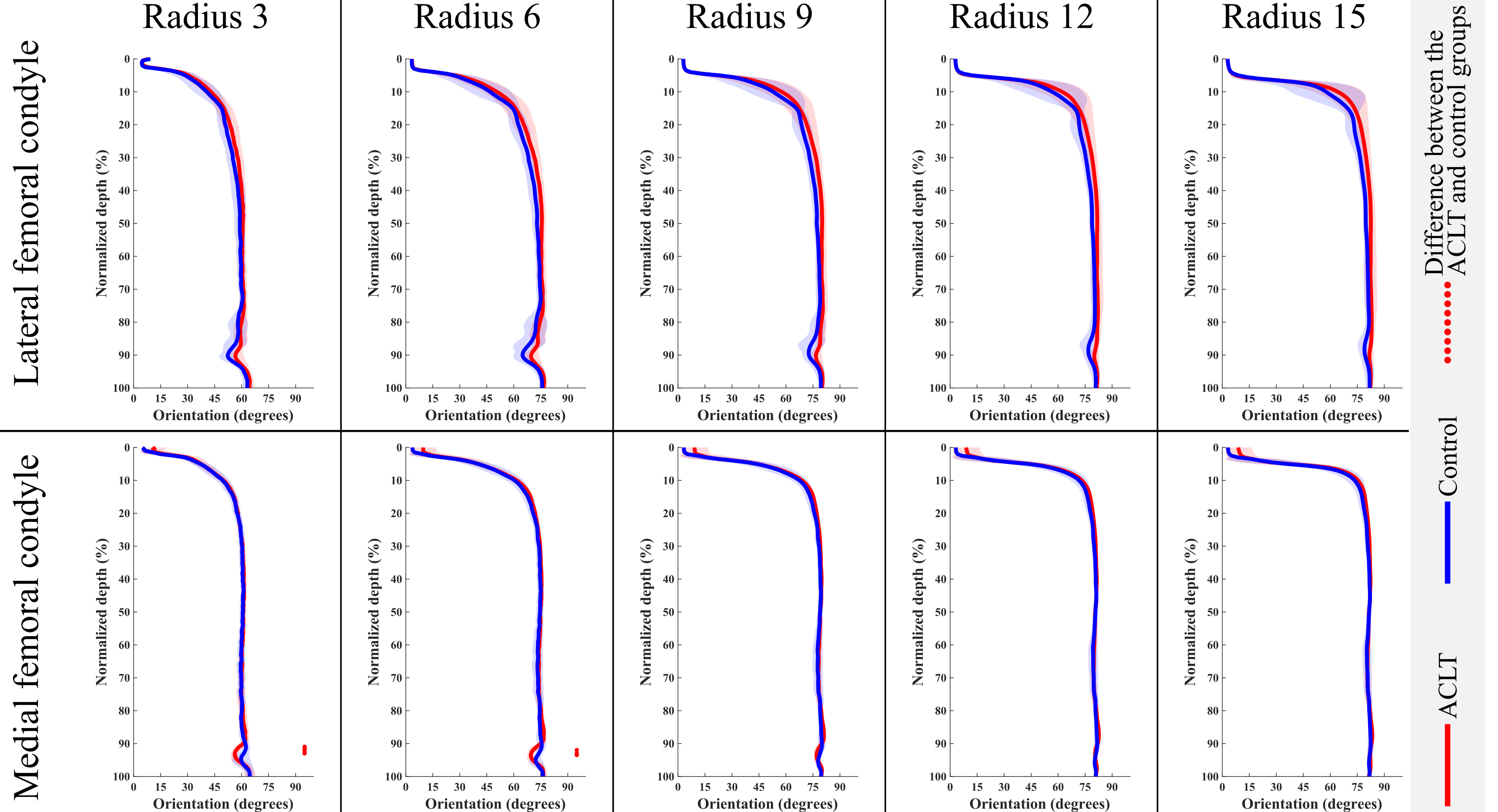

Supplement: Supplementary file 3 — Depth-wise orientation analysis of the lateral and medial femoral condyle cartilage of the healthy and the experimental groups acquired with structure tensor analysis of the µCT images (VOI: 500 µm × 500 µm × cartilage thickness) using different radius sizes (3, 6, 9, 12, 15). The data was normalized into 200 points in a depth-wise manner and each image slice (x-y-plane) was averaged laterally for a group-wise comparison. Red and blue lines represent the experimental and the healthy groups, respectively, and the shaded areas the corresponding 95% confidence intervals. The red dashed line represents the statistical difference (Mann-Whitney U-test) at the corresponding normalized depth of cartilage. Supplementary file3 (TIFF 955 kb) [file 10439_2023_3183_MOESM3_ESM.tif]

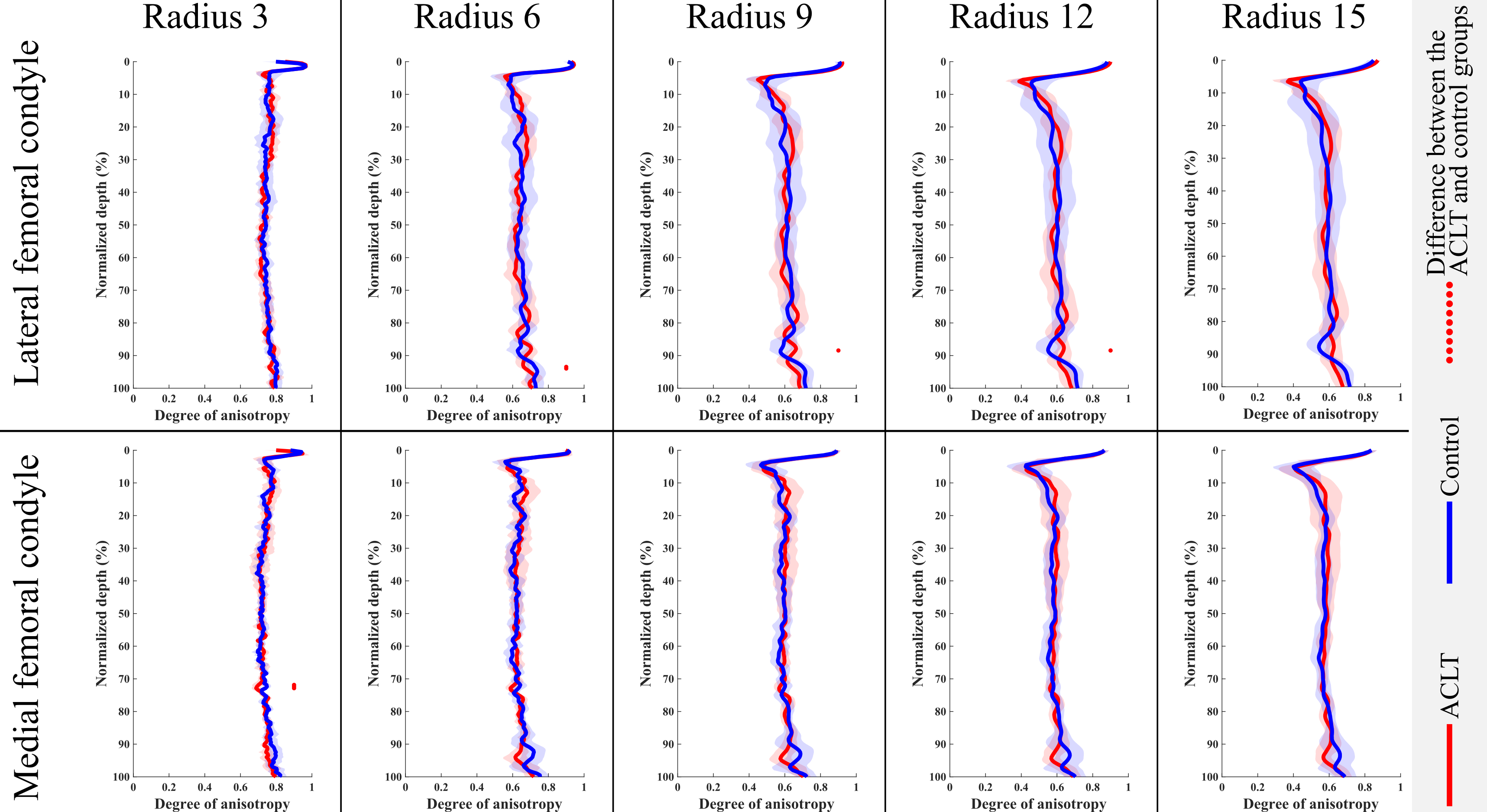

Supplement: Supplementary file 4 — Depth-wise anisotropy analysis of the lateral and medial femoral condyle cartilage of the healthy and the experimental groups acquired with structure tensor analysis of the µCT images (VOI: 150 µm × 5 µm × cartilage thickness) using different radius sizes (3, 6, 9, 12, 15). The data was normalized into 200 points in a depth-wise manner and each image slice (x-y-plane) was averaged laterally for a group-wise comparison. Red and blue lines represent the experimental and the healthy groups, respectively, and the shaded areas the corresponding 95% confidence intervals. The red dashed line represents the statistical difference (Mann-Whitney U-test) at the corresponding normalized depth of cartilage. Supplementary file4 (TIF 1031 kb) [file 10439_2023_3183_MOESM4_ESM.tif]

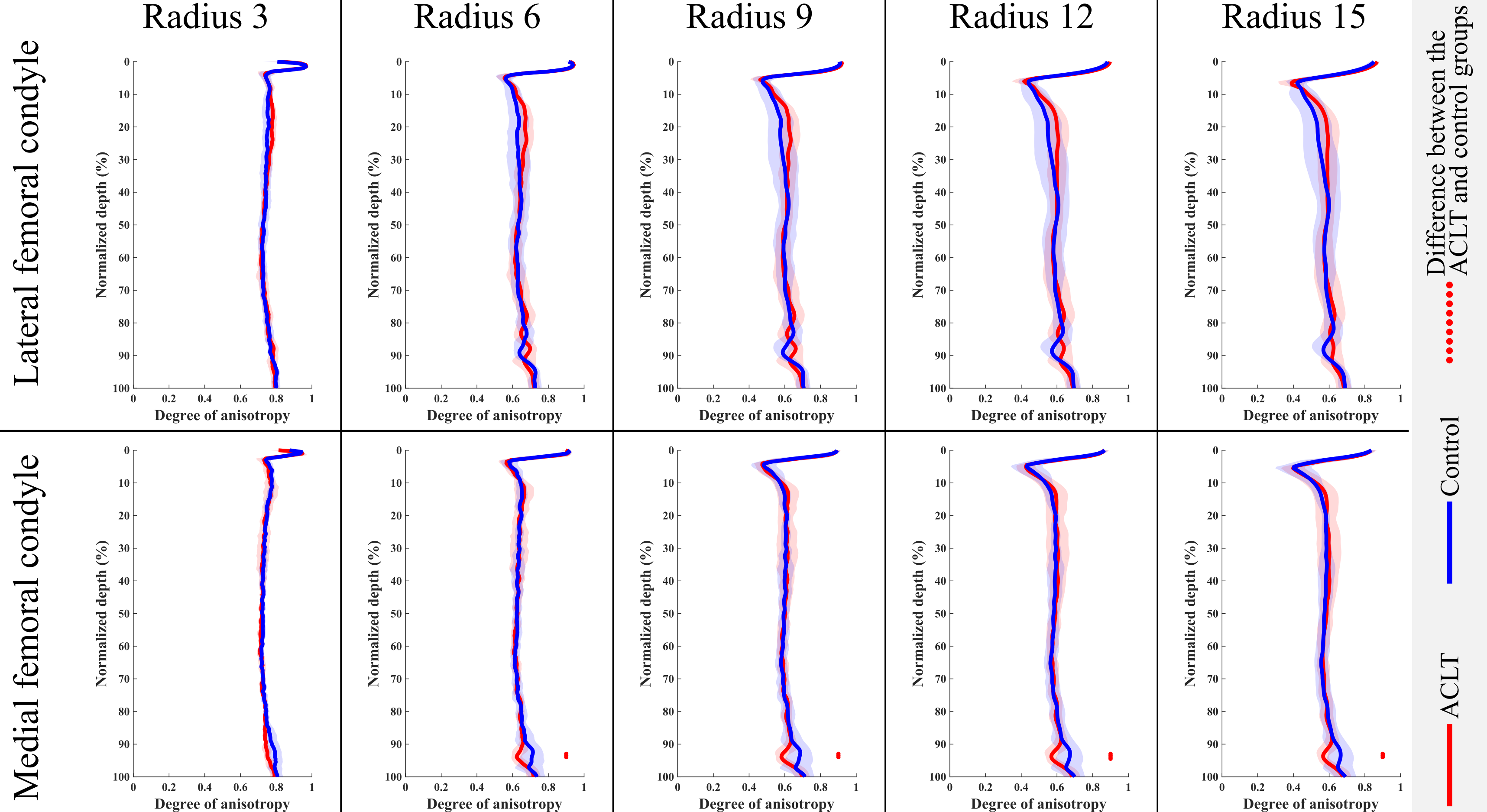

Supplement: Supplementary file 5 — Depth-wise anisotropy analysis of the lateral and medial femoral condyle cartilage of the healthy and the experimental groups acquired with structure tensor analysis of the µCT images (VOI: 150 µm × 150 µm × cartilage thickness) using different radius sizes (3, 6, 9, 12, 15). The data was normalized into 200 points in a depth-wise manner and each image slice (x-y-plane) was averaged laterally for a group-wise comparison. Red and blue lines represent the experimental and the healthy groups, respectively, and the shaded areas the corresponding 95% confidence intervals. The red dashed line represents the statistical difference (Mann-Whitney U-test) at the corresponding normalized depth of cartilage. Supplementary file5 (TIF 994 kb) [file 10439_2023_3183_MOESM5_ESM.tif]

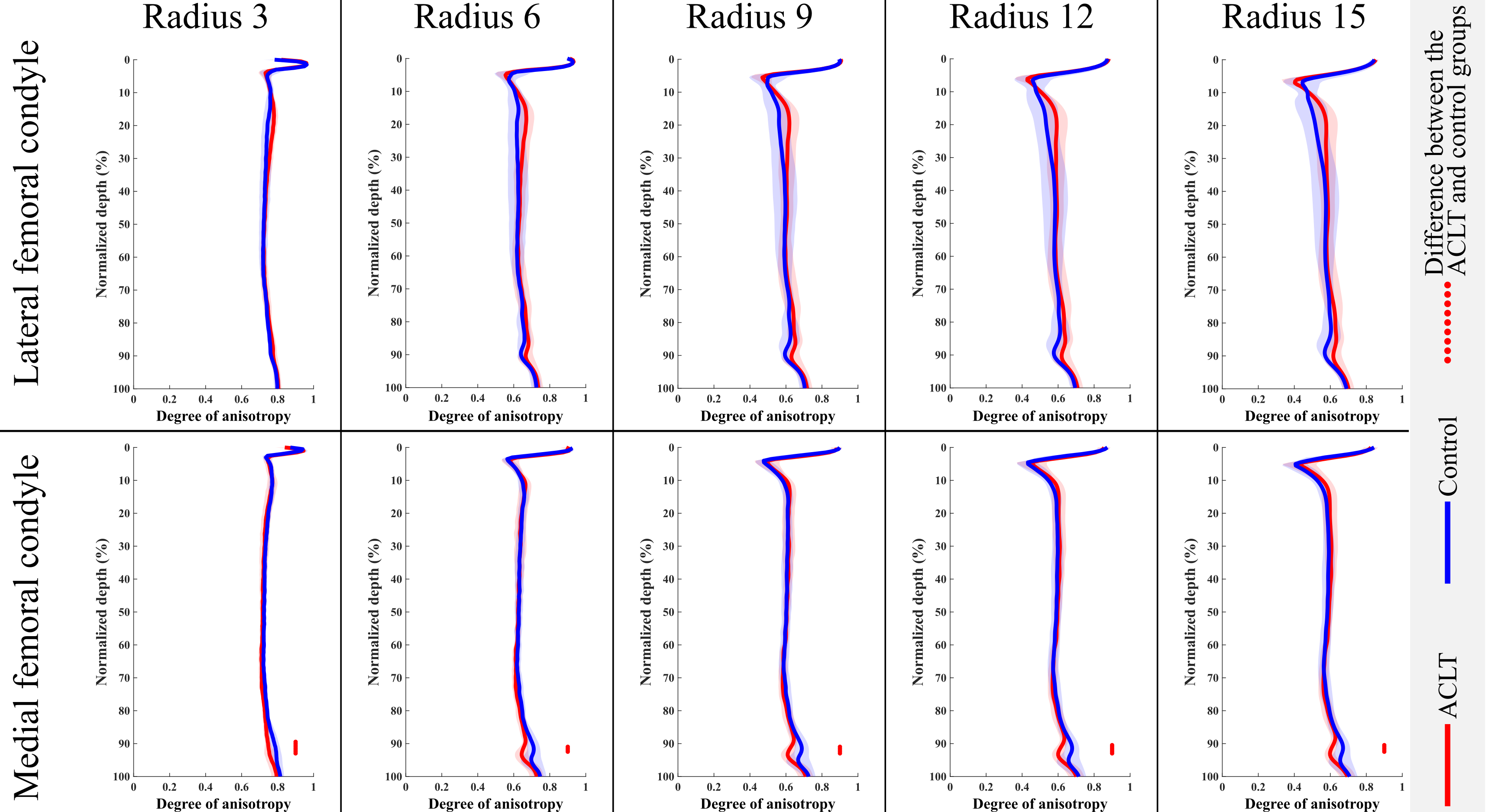

Supplement: Supplementary file 6 — Depth-wise anisotropy analysis of the lateral and medial femoral condyle cartilage of the healthy and the experimental groups acquired with structure tensor analysis of the µCT images (VOI: 500 µm × 500 µm × cartilage thickness) using different radius sizes (3, 6, 9, 12, 15). The data was normalized into 200 points in a depth-wise manner and each image slice (x-y-plane) was averaged laterally for a group-wise comparison. Red and blue lines represent the experimental and the healthy groups, respectively, and the shaded areas the corresponding 95% confidence intervals. The red dashed line represents the statistical difference (Mann-Whitney U-test) at the corresponding normalized depth of cartilage. Supplementary file6 (TIF 991 kb) [file 10439_2023_3183_MOESM6_ESM.tif]

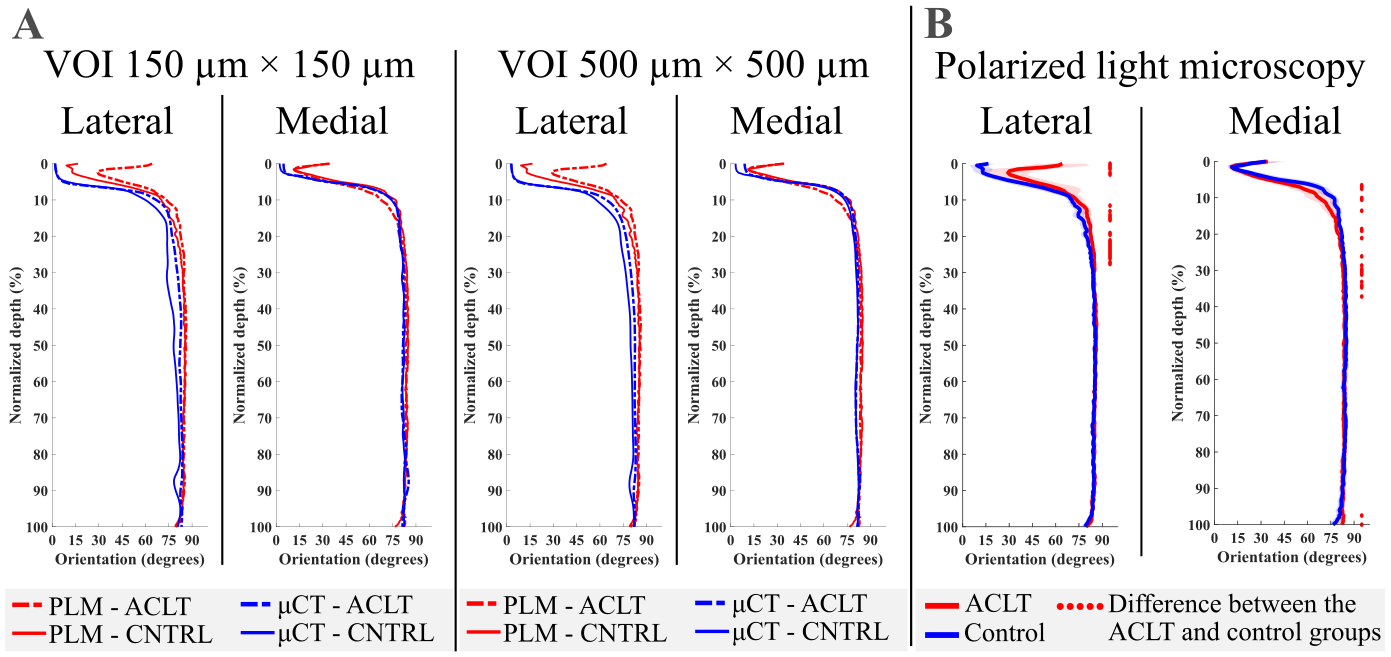

Supplement: Supplementary file 7 — Average depth-wise orientation profiles of the structure tensor analysis (integration window radius 15, VOIs: 150 µm × 150 µm × cartilage thickness, and 500 µm × 500 µm × cartilage thickness, blue lines) and the polarized light microscopy (PLM, red lines) of the lateral and medial femoral condyle cartilage of the healthy (CNTRL) and the experimental (ACLT) rabbit knee joints. B: Depth-wise orientation analysis of the lateral and medial femoral condyle cartilage of the healthy and the experimental groups acquired with polarized light microscopy. The data was normalized into 200 points in a depth-wise manner and averaged laterally for a group-wise comparison. Red and blue lines represent the experimental and the healthy groups, respectively, and the shaded areas the corresponding 95% confidence intervals. The red dashed line represents the statistical difference (Mann-Whitney U-test) at the corresponding normalized depth of cartilage. Supplementary file7 (TIF 2678 kb) [file 10439_2023_3183_MOESM7_ESM.tiff]

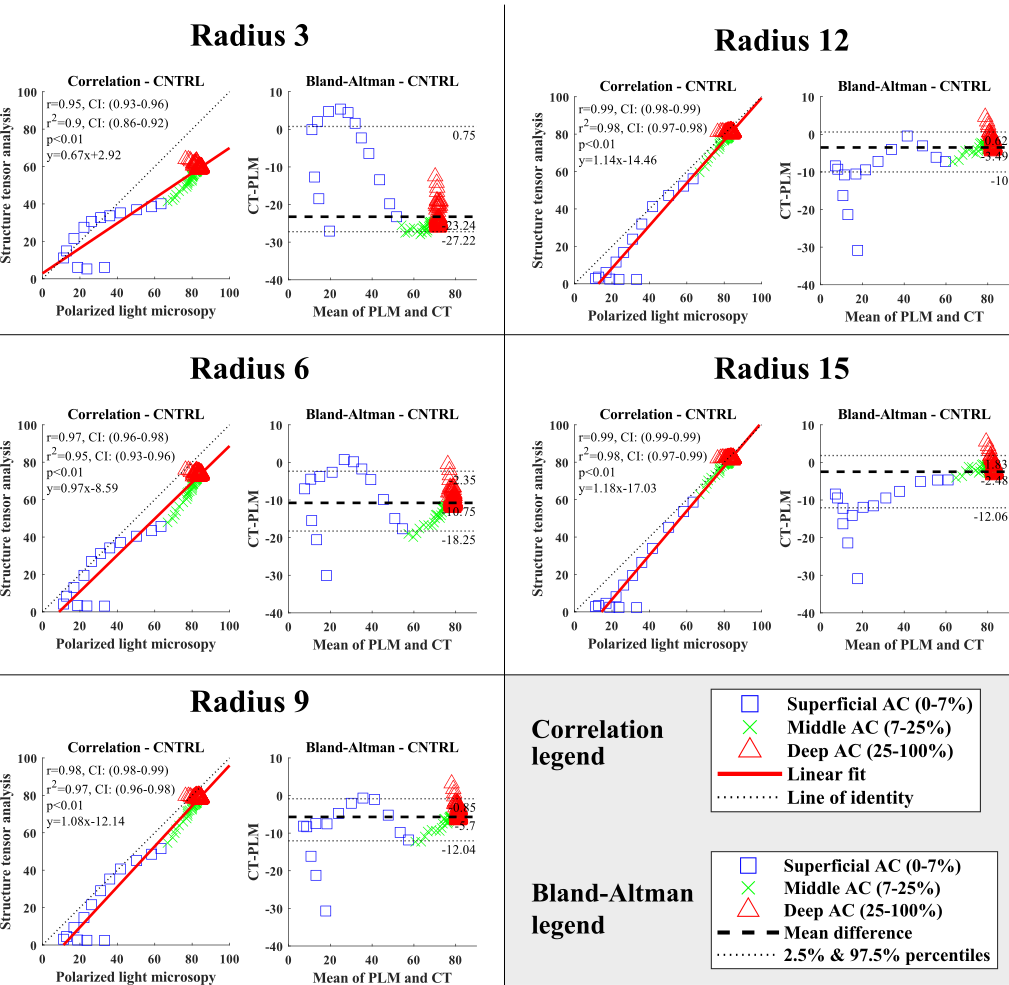

Supplement: Supplementary file 8 — The correlation and the Bland-Altman analyses of the cartilage extracellular matrix orientation acquired with polarized light microscopy (PLM) and structure tensor analysis (VOI: 150 µm × 150 µm × cartilage thickness) of the medial femoral condyle cartilage from the healthy rabbit knee joints. The analyses are made to the normalized data. The PLM analysis is compared to the different radius sizes used in the structure tensor analysis. Articular cartilage is divided into three sections in a depth-wise manner for illustrative purposes: superficial cartilage (0-7%); middle of cartilage (7-25%); deep cartilage (25-100%). This division was not used in the correlation nor Bland-Altman analyses. Supplementary file8 (TIF 2912 kb) [file 10439_2023_3183_MOESM8_ESM.tiff]

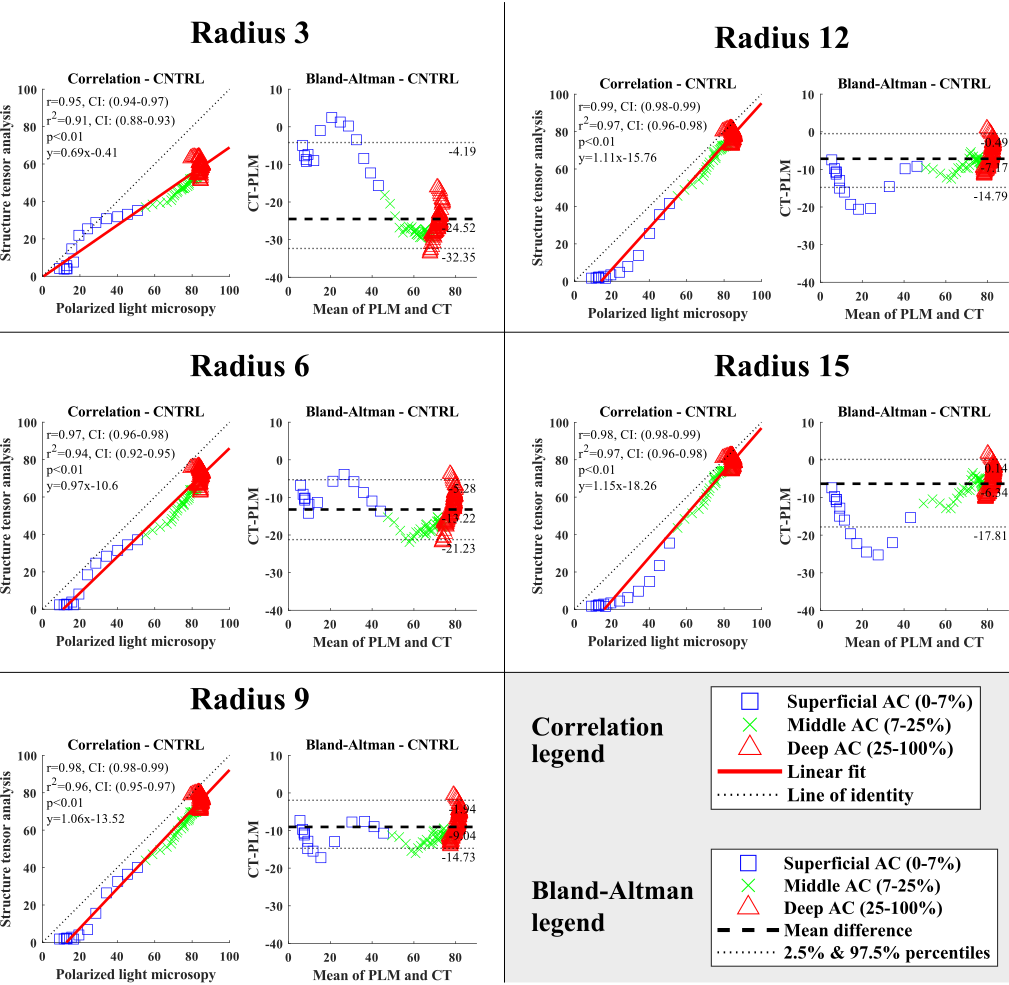

Supplement: Supplementary file 9 — The correlation and the Bland-Altman analyses of the cartilage extracellular matrix orientation acquired with polarized light microscopy (PLM) and structure tensor analysis (VOI: 150 µm × 150 µm × cartilage thickness) of the lateral femoral condyle cartilage from the healthy rabbit knee joints. The analyses are made to the normalized data. The PLM analysis is compared to the different radius sizes used in the structure tensor analysis. Articular cartilage is divided into three sections in a depth-wise manner for illustrative purposes: superficial cartilage (0-7%); middle of cartilage (7-25%); deep cartilage (25-100%). This division was not used in the correlation nor Bland-Altman analyses. Supplementary file9 (TIF 2912 kb) [file 10439_2023_3183_MOESM9_ESM.tiff]

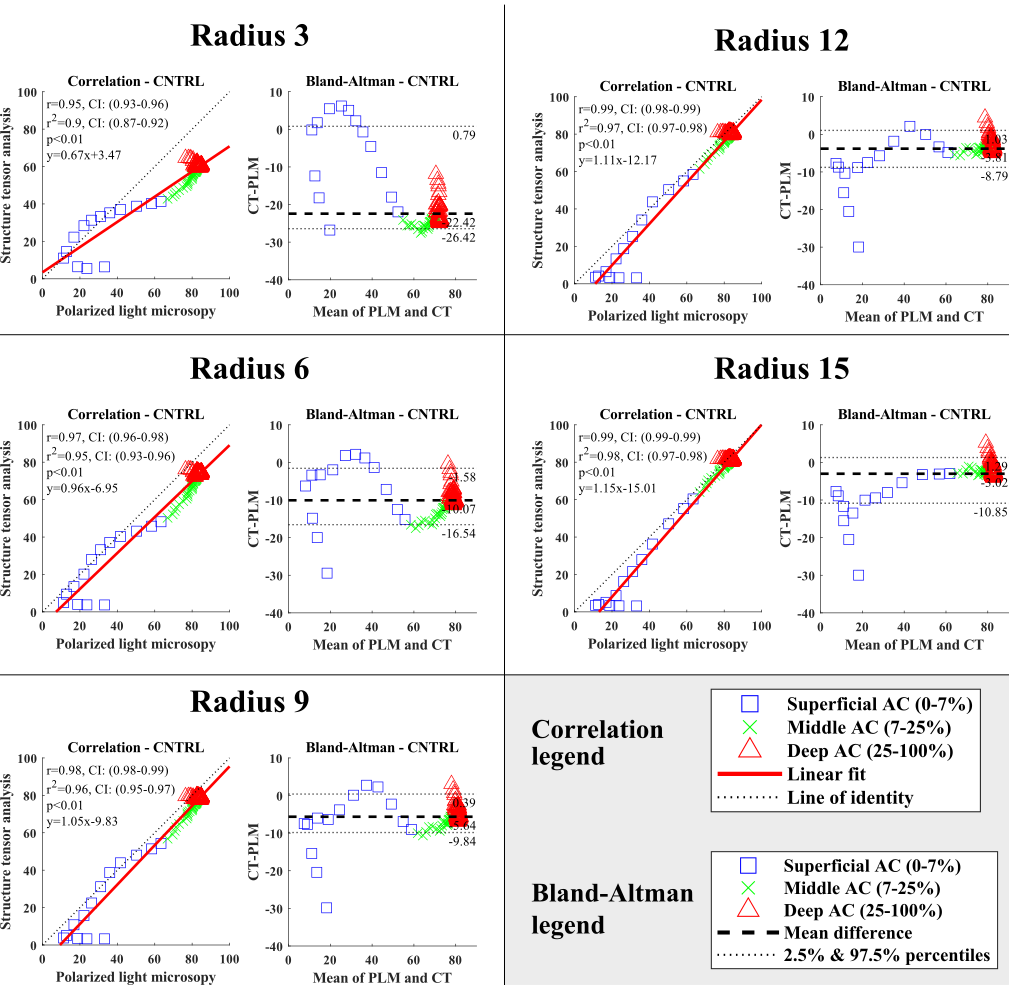

Supplement: Supplementary file 10 — The correlation and the Bland-Altman analyses of the cartilage extracellular matrix orientation acquired with polarized light microscopy (PLM) and structure tensor analysis (VOI: 500 µm × 500 µm × cartilage thickness) of the medial femoral condyle cartilage from the healthy rabbit knee joints. The analyses are made to the normalized data. The PLM analysis is compared to the different radius sizes used in the structure tensor analysis. Articular cartilage is divided into three sections in a depth-wise manner for illustrative purposes: superficial cartilage (0-7%); middle of cartilage (7-25%); deep cartilage (25-100%). This division was not used in the correlation nor Bland-Altman analyses. Supplementary file10 (TIFF 2912 kb) [file 10439_2023_3183_MOESM10_ESM.tiff]

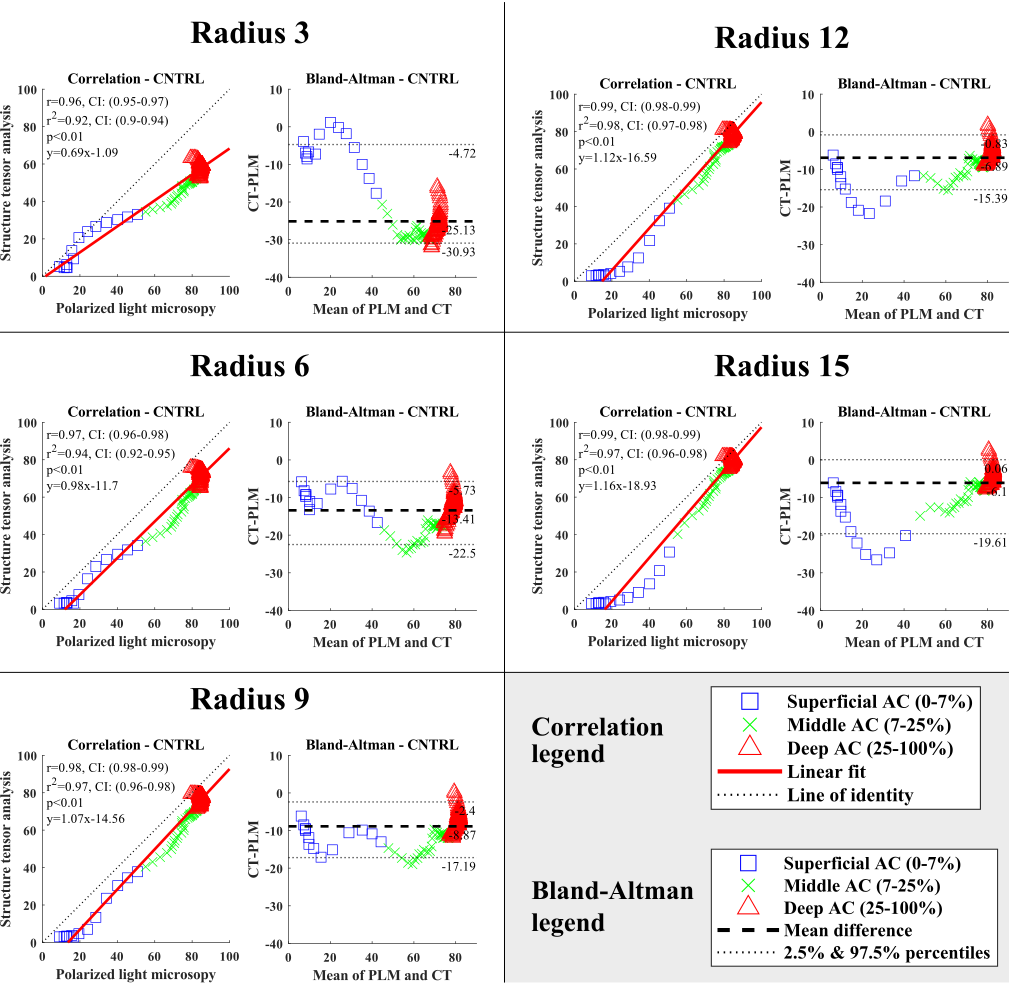

Supplement: Supplementary file 11 — The correlation and the Bland-Altman analyses of the cartilage extracellular matrix orientation acquired with polarized light microscopy (PLM) and structure tensor analysis (VOI: 500 µm × 500 µm × cartilage thickness) of the lateral femoral condyle cartilage from the healthy rabbit knee joints. The analyses are made to the normalized data. The PLM analysis is compared to the different radius sizes used in the structure tensor analysis. Articular cartilage is divided into three sections in a depth-wise manner for illustrative purposes: superficial cartilage (0-7%); middle of cartilage (7-25%); deep cartilage (25-100%). This division was not used in the correlation nor Bland-Altman analyses. Supplementary file11 (TIFF 2912 kb) [file 10439_2023_3183_MOESM11_ESM.tiff]

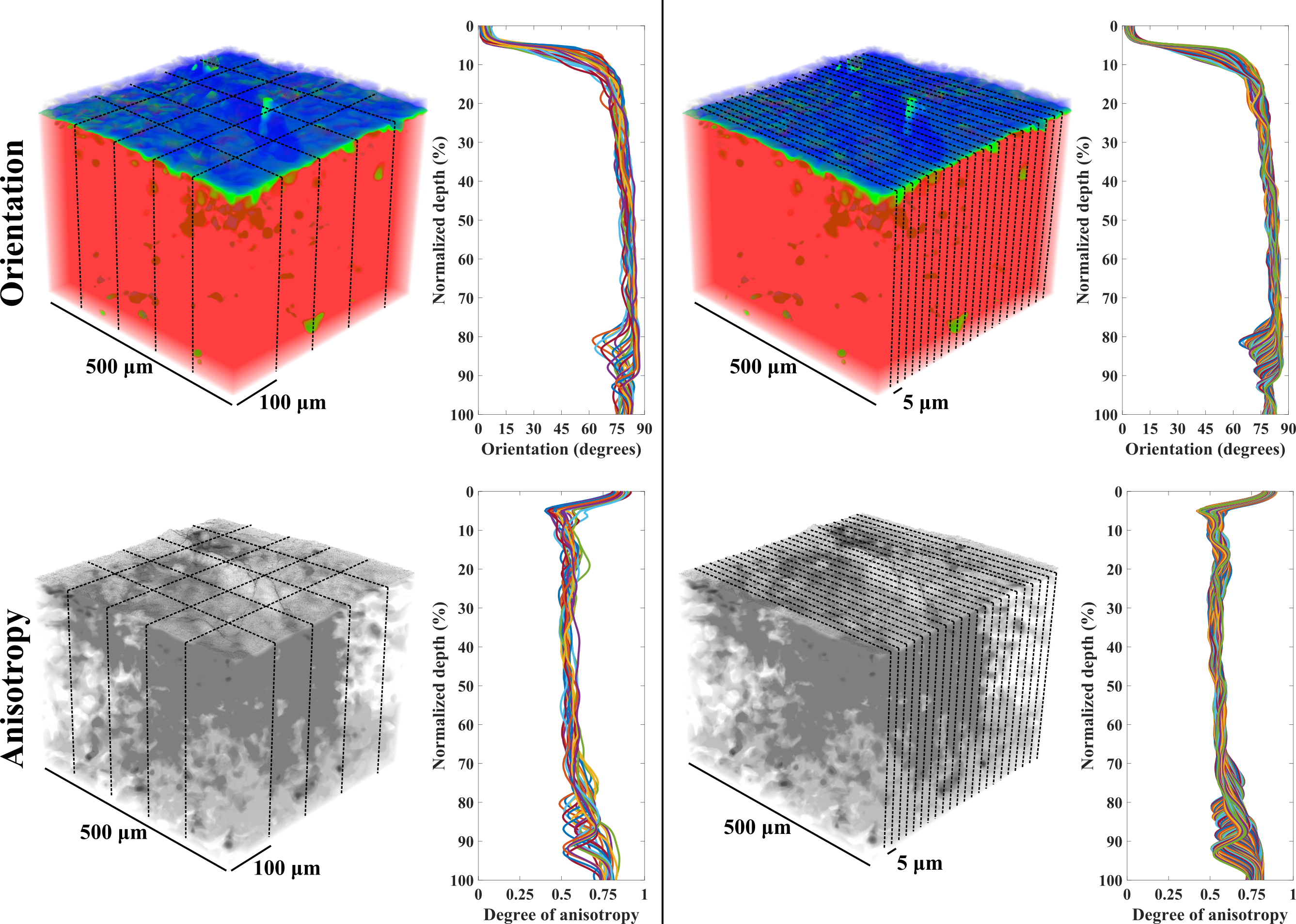

Supplement: Supplementary file 12 — Example images and depth-wise orientation and anisotropy profiles of the structure tensor analysis (integration window radius 12) of a healthy medial femoral condyle cartilage. Left: volume of 500 µm × 500 µm × cartilage thickness is laterally divided into smaller volumes of 100 µm × 100 µm × cartilage thickness and the corresponding normalized depth-wise profiles of the orientation (up) and anisotropy (down) are presented from each small volume. Right: volume of 500 µm × 500 µm × cartilage thickness is divided into smaller volumes of 500 µm × 5 µm × cartilage thickness and the corresponding normalized depth-wise profiles of the orientation (up) and anisotropy (down) are presented from each small volume. These volume divisions are not used in this study, but they are examples of some of the numerous options of the analysis regions for further investigation. Supplementary file12 (TIFF 2835 kb) [file 10439_2023_3183_MOESM12_ESM.tif]
