# Supplementary material for: Micro-computed Tomography-Based Collagen Orientation and Anisotropy Analysis of Rabbit Articular Cartilage
Source: Ann Biomed Eng. 2023 Apr 1;51(8):1769–80. doi: 10.1007/s10439-023-03183-4 (PMC10326148; doi:10.1007/s10439-023-03183-4)
Supplement: Supplementary file 13 — The Bland-Altman analyses of the cartilage extracellular matrix orientation acquired with polarized light microscopy and structure tensor analysis (VOI: 150 µm × 150 µm × cartilage thickness) of the lateral and medial femoral condyle cartilage from the healthy rabbit knee joints. Supplementary file13 (DOCX 16 kb) [file 10439_2023_3183_MOESM13_ESM.docx]

Table SI: The Bland-Altman analyses of the cartilage extracellular matrix orientation acquired with polarized light microscopy and structure tensor analysis (VOI: 150 µm × 150 µm) of the lateral and medial femoral condyle cartilage from the healthy rabbit knee joints.

| Lateral Femoral Condyle | | | | | | |
| --- | --- | --- | --- | --- | --- | --- |
| Difference (CT-PLM) | | | | | | |
| Radius | Mean | Median | STD | 2.50 % | 97.50 % | Range |
| 3 | -24.52 | -25.86 | 6.05 | -32.35 | -4.19 | 28.16 |
| 6 | -13.22 | -12.76 | 4.01 | -21.23 | -5.28 | 15.94 |
| 9 | -9.04 | -9.15 | 3.37 | -14.73 | -1.94 | 12.79 |
| 12 | -7.17 | -7.46 | 3.51 | -14.79 | -0.49 | 14.29 |
| 15 | -6.34 | -6.12 | 4.15 | -17.81 | 0.14 | 17.96 |
|  |  |  |  |  |  |  |
| Medial Femoral Condyle | | | | | | |
| Difference (CT-PLM) | | | | | | |
| Radius | Mean | Median | STD | 2.50 % | 97.50 % | Range |
| 3 | -23.24 | -24.89 | 5.62 | -27.22 | 0.75 | 27.97 |
| 6 | -10.75 | -11.11 | 3.28 | -18.25 | -2.35 | 15.90 |
| 9 | -5.70 | -5.80 | 3.00 | -12.04 | -0.85 | 11.19 |
| 12 | -3.49 | -3.46 | 3.14 | -10.00 | 0.62 | 10.62 |
| 15 | -2.48 | -2.31 | 3.48 | -12.06 | 1.83 | 13.89 |
| CT - Computed tomography | | | | | | |
| PLM - Polarized light microscopy | | | | | | |
| STD - Standard deviation | | | | | | |
| 2.50% - Value of the cumulative percentile at 2.50% | | | | | | |
| 97.50% - Value of the cumulative percentile at 97.50% | | | | | | |
| Range - 2.5% reduced from 97.5% | | | | | | |
