# Supplementary material for: Micro-computed Tomography-Based Collagen Orientation and Anisotropy Analysis of Rabbit Articular Cartilage
Source: Ann Biomed Eng. 2023 Apr 1;51(8):1769–80. doi: 10.1007/s10439-023-03183-4 (PMC10326148; doi:10.1007/s10439-023-03183-4)
Supplement: Supplementary file 14 — The Bland-Altman analyses of the cartilage extracellular matrix orientation acquired with polarized light microscopy and structure tensor analysis (VOI: 500 µm × 500 µm × cartilage thickness) of the lateral and medial femoral condyle cartilage from the healthy rabbit knee joints. Supplementary file14 (DOCX 16 kb) [file 10439_2023_3183_MOESM14_ESM.docx]

Table SII: The Bland-Altman analyses of the cartilage extracellular matrix orientation acquired with polarized light microscopy and structure tensor analysis (VOI: 500 µm × 500 µm) of the lateral and medial femoral condyle cartilage from the healthy rabbit knee joints.

| Lateral Femoral Condyle | | | | | | |
| --- | --- | --- | --- | --- | --- | --- |
| Difference (CT-PLM) | | | | | | |
| Radius | Mean | Median | STD | 2.50 % | 97.50 % | Range |
| 3 | -25.13 | -26.31 | 5.91 | -30.93 | -4.72 | 26.21 |
| 6 | -13.41 | -12.56 | 3.97 | -22.50 | -5.73 | 16.77 |
| 9 | -8.87 | -8.47 | 3.32 | -17.19 | -2.40 | 14.79 |
| 12 | -6.89 | -6.49 | 3.43 | -15.39 | -0.83 | 14.56 |
| 15 | -6.10 | -5.43 | 4.17 | -19.61 | 0.06 | 19.67 |
|  |  |  |  |  |  |  |
| Medial Femoral Condyle | | | | | | |
| Difference (CT-PLM) | | | | | | |
| Radius | Mean | Median | STD | 2.50 % | 97.50 % | Range |
| 3 | -22.42 | -23.98 | 5.56 | -26.42 | 0.79 | 27.21 |
| 6 | -10.07 | -10.22 | 3.25 | -16.54 | -1.58 | 14.96 |
| 9 | -5.64 | -5.80 | 2.90 | -9.84 | 0.39 | 10.23 |
| 12 | -3.81 | -3.94 | 2.94 | -8.79 | 1.03 | 9.82 |
| 15 | -3.02 | -2.96 | 3.18 | -10.85 | 1.29 | 12.14 |
| CT - Computed Tomography | | | | | | |
| PLM - Polarized light microscopy | | | | | | |
| STD - Standard Deviation | | | | | | |
| 2.50% - Value of the cumulative percentile at 2.50% | | | | | | |
| 97.50% - Value of the cumulative percentile at 97.50% | | | | | | |
| Range - 2.5% reduced from 97.5% | | | | | | |
